# Real-world creatine supplementation: a large-scale cross-sectional study of use, knowledge, and experiences

**DOI:** 10.1080/15502783.2026.2702952

**Published:** 2026-07-21

**Authors:** Jocelyn Burridge, Ali Boolani, Ziyang Zhang, Jordan M. Glenn

**Affiliations:** a SuppCo Inc., New York, NY, United States; b Department of Pediatrics, San Diego School of Medicine, University of California, La Jolla, CA, United States; c Human Performance and Nutrition Research Institute, Oklahoma State University, Stillwater, OK, United States; d Department of Human Physiology, Boston University, Boston, MA, United States

**Keywords:** Creatine supplementation, dietary supplements, gender differences, creatine knowledge, cross-sectional survey

## Abstract

**Background:**

Creatine is among the most extensively studied dietary supplements in sports nutrition, yet real-world supplementation patterns, knowledge, and outcomes remain poorly characterized. Prior survey research has focused on competitive athletes and younger male samples, even as the evidence base has expanded to support applications in healthy aging, cognition, and female physiology. Whether use patterns, perceived benefits, and adverse effects among the broader and more diverse population now using creatine align with trial evidence, and whether they differ by gender identity and age, has not been systematically assessed.

**Methods:**

An online cross-sectional survey was distributed to approximately 110,000 users of a supplement tracking application (SuppCo, Inc.) with recorded creatine use, yielding 2,178 analyzable responses from individuals reporting current or past-12-month creatine use (1,077 women and 1,101 men; median age, 55 years). The survey assessed supplementation patterns, goals, creatine knowledge (six-item battery scored via generalized partial credit item response theory [IRT] modeling), side effects, satisfaction, and information needs. Multivariable analyses included logistic regression for perceived benefit, modified Poisson regression for side-effect prevalence, and OLS regression for satisfaction, with Benjamini-Hochberg correction for multiple comparisons. As an exploratory analysis, a mixed graphical model (MGM) characterized conditional associations across measurement domains, and IRT discrimination parameters were combined with network centrality to identify candidate keystone misconceptions.

**Results:**

Despite 98% intending to continue supplementation and mean satisfaction of 7.8/10, only 55% perceived benefit. Cognitive performance was the most commonly endorsed goal (67.8%) yet showed the largest within-person unmet-expectation rate (64.4% of cognitive-goal endorsers did not perceive a matched cognitive benefit; McNemar *p* < .001). Women reported higher self-reported side-effect prevalence (PR = 1.48; 95% CI 1.15–1.91) and lower odds of perceived benefit (OR = 0.66; 95% CI 0.55–0.80) than men, with a flatter dose-benefit slope (women × dose OR = 0.65; 95% CI 0.53–0.80). Knowledge of creatine, scored via IRT theta from a five-item battery, was the most consistent positive correlate across all primary outcomes: perceived benefit, satisfaction, the positive experience composite, and above-common dosing. An exploratory IRT-by-network analysis identified kidney damage as the candidate keystone misconception, with the highest IRT discrimination and above-median cross-domain centrality; the network also identified long-term safety beliefs as conditionally associated with intent to continue, consistent with the hypothesis that safety beliefs track behavioral persistence.

**Conclusions:**

In this middle-aged, gender-balanced community sample of creatine users, behavioral loyalty was high yet substantial knowledge gaps, unmet benefit expectations, and self-reported side-effect burdens disproportionately affected women and older adults, who together comprise an increasingly important share of contemporary creatine users. Knowledge appeared as the most consistent positive correlate of supplementation experience, suggesting education may be a tractable lever for improving real-world outcomes; misconceptions about kidney safety and long-term tolerability may warrant particular attention as educational targets. The cognitive expectation gap may reflect a dose-translation issue rather than absence of efficacy. These findings generate hypotheses for prospective evaluation of education, gender-tailored dosing guidance, and expectation-setting interventions in real-world creatine users.

## 1. Introduction

Creatine is one of the most extensively studied dietary supplements in sports nutrition, supported by over six decades of laboratory and clinical research [1]. By expanding intramuscular phosphocreatine stores and accelerating ATP resynthesis during high-intensity effort, creatine has a well-established ergogenic effect, with meta-analyses and scoping reviews consistently demonstrating improvements in maximal strength, power output, and lean mass accrual [1–5]. The safety profile is equally well-characterised, with pooled analyses of trial data finding no difference in side-effect prevalence between creatine and placebo groups, and International Society of Sports Nutrition position statements confirming tolerability across several clinical populations [1,6,7].

A 2026 proposal to establish dietary reference intakes classifies creatine as a conditionally essential nutrient, reflecting a shift in how scientific and regulatory bodies view its use [8]. Beyond traditional applications, creatine’s roles in cellular energy buffering, mitochondrial function, and neuroprotection suggest potential benefits for cognition and healthy aging [9–13]. In recent years, creatine has become widely accessible and is now used by a broader population than the young, active men who historically dominated clinical trials and survey research [2,9,12,14,15].

This expansion into new populations is supported by a growing clinical evidence base. In older adults, creatine combined with resistance training improves lean mass, bone mineral density, and functional capacity, supporting applications for age-related osteoporosis and frailty [11,13,16]. Cognitive applications have also gained attention, with meta-analyses reporting small but significant benefits for memory and processing speed [7,17–19]. However, subgroup analyses suggest these benefits are greatest under conditions of energetic demand or low baseline creatine availability, including in older adults, females, vegetarians, and sleep-deprived individuals, with effects attenuating considerably in well-rested, healthy adults [18,19]. Female physiology is therefore of particular relevance to both the musculoskeletal and cognitive applications. Women generally have lower endogenous creatine stores than men, estimated at roughly 70 to 80 percent of male levels [20]. Life stage transitions such as menstruation, pregnancy, and menopause may alter creatine physiology and potentially increase responsiveness to supplementation [20–22]. Despite this, dosing guidance has been derived largely from male samples, even as reviews have called for research on optimal thresholds and dose–response patterns across the female lifespan [2,23–25].

Although these benefits are well supported under controlled conditions, it remains unclear how well they translate to contemporary use and whether users perceive similar effects. Most real-world studies have focused on competitive athletes, bodybuilders, or organised sports supplement users, limiting generalisability to broader populations [26–29]. The few community-based or consumer-facing surveys that exist report variable use rates (6–28%), primary reliance on non-professional information sources, and frequent gaps in dosing knowledge, though they have generally been restricted to narrow populations or single outcome domains [14,30,31]. In parallel, concerns about kidney damage, hair loss, and dehydration are not supported by evidence, yet little empirical work has quantified the prevalence of these misconceptions, examined variation by gender identity and age, or identified which are most actionable for educational intervention [32,33].

Despite the growing clinical evidence base, fundamental questions about real-world creatine use remain unresolved. Prior real-world studies have largely focused on athletic or fitness-focused samples and do not adequately assess whether use patterns, knowledge, perceived benefits, and adverse effects differ by gender and age or align with effects documented in controlled trials. The present study addresses these gaps in a large, demographically diverse sample of current and recent creatine users by characterising dosing and duration patterns, goals for use, knowledge and misconceptions, self-reported side effects, satisfaction, and user narratives, with analyses stratified by gender identity and age.

## 2. Methods

### 2.1. Study design and participants

This cross-sectional online survey characterised creatine supplementation behaviours, knowledge, experiences, and perceived benefits among adults currently using creatine or who had used it in the past 12 months. Eligible participants were adults aged 18 or older who responded affirmatively to either “currently taking creatine” or “yes, but not currently” on the eligibility screen. The survey was distributed via the SuppCo platform (SuppCo, Inc.; New York, New York), a supplement tracking and consumer education application, to the approximately 110,000 platform users who were identified as logging creatine on the platform at the time of dissemination, using a combination of in-app notifications, e-mail, and other digital communications. A total of 2,233 individuals completed the survey (approximate completion rate 2%).

**Figure 1.**
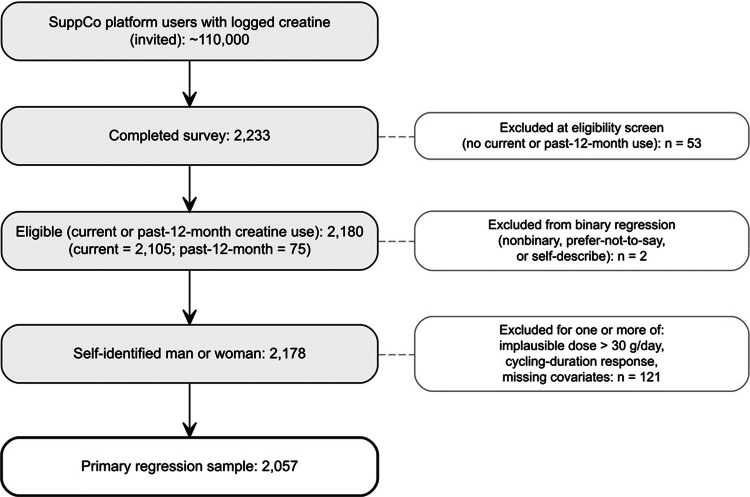
STROBE-style sample flow. Flow of survey responses from platform invitation through the primary regression analytic sample, with side annotations documenting each exclusion. From approximately 110,000 SuppCo platform users who had been identified as logging creatine on the platform, 2,233 individuals completed the survey (~2% completion rate among those eligible to receive the invitation); 53 were excluded as not currently or past-12-month users; 55 were excluded as nonbinary, prefer-not-to-say, or self-describe (retained in descriptive summaries); and 121 were excluded as cycling-duration responses, implausible dose > 30 g, or missing covariates, leaving 2,057 in the primary regression analytic sample.

Of these, 2,180 met eligibility for the analytic cohort by reporting current creatine use (*n* = 2,105; 96.6% of the eligible cohort) or use within the past 12 months but not currently (*n* = 75; 3.4%); the remaining 53 respondents (2.4% of completers) did not endorse either option and were excluded at the eligibility screen. Past-12-month users were asked to respond about their most recent regimen, and a sensitivity analysis confirmed that excluding past-12-month users did not change the direction or significance of any primary effect. No financial incentives were offered. The survey was administered as a de-identified instrument. After completing the survey, participants were given the optional opportunity to provide an email address to receive a summary of findings; this field was separate from the survey instrument, was not linked to survey responses, and was not included in the analytic dataset. Sample flow is depicted in Figure 1.

### 2.2. Survey instrument and measures

The survey instrument captured seven measurement domains: (1) demographics, (2) supplementation patterns, (3) goals and perceived benefits, (4) creatine knowledge, (5) side effects, (6) satisfaction and continuation, and (7) free-text information needs.

**Demographics.** Participants reported date of birth, gender identity (man, woman, nonbinary, prefer not to say, self-describe), height, and body weight. The survey did not collect sex assigned at birth; the available variable is therefore self-reported gender identity, and we use “men”/“women” throughout the body text. For binary regression models, “man” and “woman” responses were retained (*n* = 2,178), and the 55 respondents identifying as nonbinary, prefer-not-to-say, or self-describe were retained in descriptive summaries but excluded from binary regression models. Anthropometric values outside plausibility bounds were set to missing (*n* = 1). Ages below 18 or above 100 years were set to missing (*n* = 38). Activity level was assessed on a five-category ordinal scale (sedentary, lightly active, moderately active, highly active, competitive or professional athlete).

**Supplementation patterns.** Duration of current creatine use included six ordered categories (<1 month through 3+ years) plus a non-ordinal option (“I cycle on and off, unsure of total duration”), which was excluded from regression models and retained in descriptive summaries. Dose information was recorded as daily dose (g), use of a loading phase (yes, no, unsure), and dosing frequency. Implausible daily doses were excluded (*n* = 7; reported values ranged from 750 to 2,500 g/day). Dose was categorised as: low (<3g/day), below common (3–4g/day), common (5g/day), elevated (6–10g/day), high (11–30g/day). Throughout the manuscript, we describe doses >5 g/day as “above-common,” relative to the modal 5 g/day reported in this sample and 3–5 g/day maintenance dose commonly referenced in the creatine literature. This terminology is intended to describe dosing relative to a common fixed-dose maintenance approach, not to imply that doses >5 g/day are inherently excessive or unsafe. For context, the ISSN position stand describes weight-based maintenance dosing of approximately 0.1 g/kg/day (~5–10 g/day for a 50–100 kg individual), with an optional loading phase of 0.3 g/kg/day (~15–30 g/day) for 5–7 days [1].

Participants also reported their primary creatine form by selecting one or more of: monohydrate, micronized monohydrate, HCl, buffered, nitrate, multi-ingredient blend, “not sure,” or other. The form distribution is reported in Table 2.

**Goals for use and perceived benefits.** Participants endorsed goals from a checklist of 11 options (muscle strength, muscle mass, exercise performance, exercise recovery, injury recovery, cognitive performance, energy/fatigue reduction, healthy aging, weight management, general wellness, medical/clinical use). Overall perceived benefit was assessed as a single item (yes, unsure, no). Participants perceiving benefit identified specific benefit domains (strength, muscle mass, exercise volume/endurance, recovery, cognition, mood, energy).

**Creatine knowledge.** Five statements assessed common beliefs about creatine, informed by frequently cited misconceptions in the creatine literature [32]. Participants indicated whether each was true, false, or “unsure.” The statements were: (1) creatine causes kidney damage (correct answer: false); (2) creatine causes hair loss (false); (3) creatine is a steroid (false); (4) creatine is only beneficial for weightlifters (false); and (5) long-term creatine use is unsafe (false). A sixth water-retention item was administered but excluded from the primary analysis: the item wording conflated a transient loading-phase hydration mechanism with the colloquial long-term-retention misconception, and respondents could endorse the statement under either interpretation. This exclusion made no substantive difference to the results. For descriptive analyses, a simple unweighted sum score (range 0–5) was computed based on the number of correct responses.

**Side effects.** Participants reported whether they had experienced any side effects from creatine supplementation (yes or no). Specific side effects assessed included: stomach upset, bloating, water retention, weight gain, and muscle cramps.

**Satisfaction and loyalty.** Overall satisfaction with creatine was rated on a 10-point scale (1–10). Intent to continue creatine supplementation over the next six months was assessed as yes/no/unsure.

**Free-text responses.** Participants completed an open-ended item: “What do you wish you had known about creatine before you started?”

**Co-supplements.** Participants indicated which of 11 commonly co-administered supplements they were currently using alongside creatine (protein powder, pre-workout, beta-alanine, citrulline, electrolytes, omega-3, multivitamin, vitamin D, magnesium, caffeine, and nootropics).

### 2.3. Statistical analysis

Across analyses, “unsure” responses were handled consistently. For binary regression modelling, “unsure” responses to the perceived-benefit and intent-to-continue items were combined with “no.” For the knowledge items, the polytomous generalised partial credit model (GPCM) retained all three response categories (incorrect, unsure, correct) as ordered, while the 2PL binary model used for the keystone-misconception discrimination analysis collapsed “unsure” with “incorrect.” Missing covariates were handled by listwise deletion within each regression model; analytic sample sizes for each model are reported in the Model Specification Table (Supplementary Table S8).

#### 2.3.1. Descriptive statistics and comparisons

Continuous variables are summarised as mean (standard deviation [SD]) and median (interquartile range [IQR]), and categorical variables are presented as frequencies and percentages. Gender differences were evaluated using Wilcoxon rank-sum tests (continuous/ordinal) and chi-squared tests (categorical); age group comparisons used Kruskal–Wallis and chi-squared tests. Effect sizes were quantified using rank-biserial |r| and Cramér’s V for chi-squared tests. To control the false discovery rate, *p*-values were adjusted using the Benjamini-Hochberg (BH) procedure applied separately within each hypothesis family (bivariate gender comparisons, 12 tests; goal endorsement models, 22 tests; benefit-type models, 36 tests; knowledge item models, 36 tests; q < 0.05).

#### 2.3.2. IRT modelling and diagnostics

A generalised partial credit model (GPCM) was fitted to the five polytomous knowledge items (incorrect, unsure, correct) to derive latent knowledge scores for multivariable analyses. Model parameters were estimated using marginal maximum likelihood via the expectation-maximisation (EM) algorithm [34,35]. Expected a posteriori (EAP) estimates of the latent knowledge trait (*θ*) were derived and used as the primary knowledge score in all multivariable models. Model fit was evaluated using M2, RMSEA, and marginal reliability [36].

The five-item GPCM showed acceptable fit (M₂(5) = 6.58, *p* = 0.254; RMSEA = 0.012; SRMSR = 0.039; CFI = 0.999; marginal reliability = 0.475). Because marginal reliability is modest, IRT-theta scores are best interpreted at the group level (e.g. as covariates in regression) rather than as precise individual diagnostics; we therefore also report sensitivity analyses with a standardised 0–5 sum score in Supplementary Table S6. A separate 2PL model was fitted to binary responses (correct vs. incorrect/unsure) to obtain item discrimination parameters used in the exploratory keystone misconception analysis [34–36].

#### 2.3.3. Perceived benefit models

Overall perceived benefit (binary; “unsure” combined with “no”) was modelled using logistic regression (*N* = 2,057) with predictors: age, gender (man/woman; binary), daily dose in 5-g units, duration of use (ordinal), activity level (ordinal), knowledge *θ* in IRT-theta SD units, and number of co-supplements. Self-reported side-effect history and number of goals endorsed were excluded to avoid collider bias and mechanical coupling, respectively. Model stability was evaluated using bootstrap validation with 2,000 resamples. A pre-specified gender × dose interaction was evaluated in an extended model; continuous predictors were mean centred for interpretability.

To examine the shape of the dose–benefit association, GAMs with smooth spline terms for dose and age were fitted adjusting for gender identity and knowledge *θ* score.

#### 2.3.4. Goal endorsement models

Each of 11 goals was modelled using logistic regression with age (per 10-year increase) and gender identity (man/woman) as predictors. BH-FDR correction was applied across 22 tests. To compare goal endorsement with the matched perceived-benefit item within respondents, we computed, for each of the five goal–benefit pairs available, the conditional proportion of goal-endorsers who reported the matched perceived benefit and tested the null that the paired discordant counts were equal using McNemar’s exact-binomial test. Results of these within-person paired comparisons are reported in Table 4.

#### 2.3.5. Benefit-domain model

Each of six benefit domains was modelled as a binary outcome using logistic regression adjusted for age, gender identity, dose, duration, activity, and knowledge *θ*, and number of co-supplements, with BH-FDR correction.

#### 2.3.6. Side-effect model

Prevalence ratios for self-reported any side effect were estimated using modified Poisson regression with HC0 robust standard errors (“unsure” combined with “no” for the binary side-effect indicator), with predictors: age, gender identity, daily dose in 5-g units, duration of use, activity level, knowledge *θ*, loading phase use, and number of co-supplements.

#### 2.3.7. Satisfaction and positive experience composite

Satisfaction (1–10 scale) was modelled using OLS regression, the primary model excluded perceived benefit to reduce endogeneity (perceived benefit shares measurement error with satisfaction). Predictors: age, gender identity, dose in 5-g units, duration, activity, knowledge *θ*, side-effect history, number of co-supplements, and number of goals.

A positive experience composite (perceived benefit + satisfaction ≥8 + intent to continue) was modelled using logistic regression with age, gender identity, daily dose per 5 g, duration of use, activity level, knowledge in IRT-theta SD units (harmonised with the other primary models), number of goals endorsed, and number of co-supplements.

#### 2.3.8. Above-common dosing behaviour

Above-common dosing (>5 g/day; relative to the modal sample dose) was modelled using logistic regression with age, gender, duration, activity, knowledge *θ*, number of goals endorsed, prior loading phase use, and number of co-supplements.

#### 2.3.9. Free-text thematic analysis

Responses to the open-ended item “What do you wish you had known about creatine before you started?” were analysed using a semi-automated hybrid coding approach. Themes were developed deductively from creatine and sports nutrition education literature and refined inductively by reviewing a random 20% subsample of responses. Each response could be assigned multiple thematic codes (themes were not mutually exclusive). Responses were matched to themes using regular expression patterns applied to lowercased text strings; all matches were reviewed for false positives by JB, who also audited a random sample of untagged responses for false negatives and reclassified missed themes. To assess inter-rater reliability, JMG independently coded a 15% random subsample of responses; agreement was high (Cohen’s *κ* = 0.837 across the seven domains), and discrepancies were resolved by discussion. Themes were organised into seven higher-order information-need domains: Benefits Awareness, Protocol Guidance, Myth Correction, Side Effects and Body Changes, Practical Advice, Expectation Framing, and Wish Started Sooner. Responses containing trivial entries (e.g. “N/A”) were excluded.

#### 2.3.10. Cross-domain network analysis (exploratory)

A mixed graphical model (MGM) was fitted using ℓ1-penalised (LASSO) neighbourhood regression, treating binary and nominal variables as Bernoulli-distributed and ordinal and continuous variables as Gaussian approximations. The LASSO penalty parameter was tuned via the Extended Bayesian Information Criterion (EBIC) with *γ* = 0.25. The resulting network included nodes from all seven domains: knowledge items (5), goals (11), side effect types (5), demographics (3), supplementation patterns (4), outcomes (3), and free-text themes meeting a ≥5% endorsement threshold. Nodes with zero variance were excluded prior to fitting.

Community structure was assessed using the walktrap random-walk algorithm. Node strength and bridge centrality were calculated to identify highly connected nodes and cross-domain connectors. As an exploratory exercise, IRT discrimination parameters were considered alongside network centrality; knowledge items with both above-median discrimination and centrality were classified as candidate “keystone misconceptions” warranting further investigation. This framework is exploratory and hypothesis-generating, requiring replication with larger item batteries and prospective designs before informing intervention priorities.

Stability of edge weights was assessed by non-parametric bootstrap (B = 200; Supplementary Table S7); stability of strength centrality ordering was assessed by case-dropping bootstrap and summarised with the CS-coefficient (Supplementary Figure S6). Edges with bootstrap inclusion below 65% are flagged as unstable in the supplement.

### 2.4. Software

All analyses were conducted in R version 4.4.1 (R Foundation for Statistical Computing; RStudio 2024.04.1 + 748).

### 2.5. Ethical considerations

All individuals provided electronic informed consent prior to participation. Recruitment procedures and study protocols were approved by Oklahoma State University’s Institutional Review Board (Stillwater, OK, USA).

## 3. Results

### 3.1. Participant characteristics overall and by gender identity

A total of 2,233 individuals completed the survey; 2,180 met eligibility (2,105 currently using creatine, 75 used in the past 12 months but not currently) and 2,178 self-identified as a man or a woman (Figure 1; STROBE-style sample flow). The 55 respondents identifying outside the binary man/woman categories (nonbinary, prefer-not-to-say, or self-describe; per-category counts shown in the Table 1 footnote) were retained in descriptive demographic summaries and excluded from binary regression models because of insufficient subgroup size for stable adjusted estimates. Regression analyses used a subset of 2,057 respondents, after excluding cycling-duration responses (no ordinal rank), implausible-dose responses (*n* = 7), and those with missing covariates.

**Table 1.** Participant characteristics.

Characteristic	Overall *N* = 2,178	Men *N* = 1,101	Women *N* = 1,077	*p*-value
**Age (years)**				0.007
Mean (SD)	54 (12)	53 (13)	55 (11)	
Median (Q1, Q3)	55 (46, 63)	54 (44, 63)	56 (47, 64)	
(Missing)	37	19	18	
**BMI (kg/m2)**				<0.001
Mean (SD)	28 (6)	29 (6)	26 (6)	
Median (Q1, Q3)	26 (23, 31)	27 (25, 31)	25 (22, 30)	
(Missing)	128	107	21	
**Daily dose (g)**				<0.001
Mean (SD)	6.72 (3.20)	7.50 (3.64)	5.93 (2.42)	
Median (Q1, Q3)	5.00 (5.00, 10.00)	5.00 (5.00, 10.00)	5.00 (5.00, 5.00)	
(Missing)	7	1	6	
**Activity level**				0.5
Sedentary	43 (2.0%)	21 (1.9%)	22 (2.0%)	
Lightly active	197 (9.0%)	88 (8.0%)	109 (10%)	
Moderately active	938 (43%)	479 (44%)	459 (43%)	
Highly active	981 (45%)	502 (46%)	479 (44%)	
Competitive athlete	19 (0.9%)	11 (1.0%)	8 (0.7%)	
**Age group**				<0.001
18-29	69 (3.2%)	47 (4.3%)	22 (2.1%)	
30-39	201 (9.4%)	127 (12%)	74 (7.0%)	
40-49	505 (24%)	251 (23%)	254 (24%)	
50-59	612 (29%)	292 (27%)	320 (30%)	
60-69	583 (27%)	275 (25%)	308 (29%)	
70+	171 (8.0%)	90 (8.3%)	81 (7.6%)	
(Missing)	37	19	18	

^1^ n (%); ^2^ Wilcoxon rank sum test; Pearson’s Chi-squared test. Note: Of 2,233 total completed surveys, 55 respondents reported a gender identity outside the binary categories (1 nonbinary, 1 prefer-not-to-say, 53 self-describe); these respondents were excluded from this table because the subgroup was too small for stable adjusted comparisons; these cases partly overlap with respondents excluded on other criteria.

Creatine form distributions are reported in Table 2: the most commonly endorsed form was monohydrate or micronized monohydrate (which is monohydrate processed for finer particle size, not a chemically distinct compound); 95.4% of respondents endorsed monohydrate and/or micronized, and only 4.0% selected exclusively non-monohydrate forms (HCl, buffered, nitrate, blends, or “not sure”; full breakdown in Table 2). Most respondents reported low concern about creatine safety (71.7% not at all concerned, 20.3% slightly concerned, 8.0% moderately to extremely concerned) and moderate self-rated confidence in their understanding of creatine (43.2% moderately confident, 16.4% very confident, 4.5% extremely confident, 27.7% slightly confident, 8.2% not at all confident). The most commonly endorsed primary sources from which respondents first learned about creatine were research articles or scientific sources, healthcare professionals, and social media or influencers.

**Table 2.** Patterns of creatine supplement use and dosing practices.

Characteristic	Overall *N* = 2,178	Men *N* = 1,101	Women *N* = 1,077	*p*-value
**Duration of use**				**<0.001**
<1 mo	52 (2.4%)	26 (2.4%)	26 (2.4%)	
1-3 mo	183 (8.4%)	66 (6.0%)	117 (11%)	
3-6 mo	283 (13%)	109 (9.9%)	174 (16%)	
6-12 mo	550 (25%)	231 (21%)	319 (30%)	
1-3 yr	645 (30%)	301 (27%)	344 (32%)	
3+ yr	381 (17%)	306 (28%)	75 (7.0%)	
Cycling	84 (3.9%)	62 (5.6%)	22 (2.0%)	
**Daily dose (g/day, < = 30g)**				**<0.001**
Mean (SD)	6.72 (3.20)	7.50 (3.64)	5.93 (2.42)	
Median (Q1, Q3)	5.00 (5.00, 10.00)	5.00 (5.00, 10.00)	5.00 (5.00, 5.00)	
(Missing)	7	1	6	
**Dose category**				
Low (<3g)	43 (2.0%)	18 (1.6%)	25 (2.3%)	
Below common (3-4g)	78 (3.6%)	23 (2.1%)	55 (5.1%)	
Common (5g)	1,304 (60%)	561 (51%)	743 (69%)	
Elevated (6-10g)	628 (29%)	405 (37%)	223 (21%)	
High (11-30g)	118 (5.4%)	93 (8.4%)	25 (2.3%)	
Implausible (>30g)	7 (0.3%)	1 (<0.1%)	6 (0.6%)	
**Creatine form (primary classification)**				**<0.001**
Monohydrate only	1,505 (69%)	748 (68%)	757 (70%)	
Micronized only	456 (21%)	233 (21%)	223 (21%)	
Monohydrate + micronized	118 (5.4%)	83 (7.5%)	35 (3.2%)	
Non-monohydrate only (HCl/buffered/nitrate/blend/unsure)	88 (4.0%)	29 (2.6%)	59 (5.5%)	
Not reported	11 (0.5%)	8 (0.7%)	3 (0.3%)	
**Loading phase**				**<0.001**
No	1,616 (74%)	813 (74%)	803 (75%)	
Not sure what a loading phase is	210 (9.6%)	66 (6.0%)	144 (13%)	
Yes	352 (16%)	222 (20%)	130 (12%)	
**Dosing frequency**				**<0.001**
Only during training phases	24 (1.1%)	17 (1.5%)	7 (0.6%)	
Inconsistently	30 (1.4%)	14 (1.3%)	16 (1.5%)	
A few times per week	121 (5.6%)	48 (4.4%)	73 (6.8%)	
Once daily	1,723 (79%)	823 (75%)	900 (84%)	
Multiple times daily	280 (13%)	199 (18%)	81 (7.5%)	

^1^n (%); ^2^Pearson’s Chi-squared test; Wilcoxon rank sum test; NA. Note: Creatine form classification: respondents could endorse multiple forms in the survey. The Non-monohydrate-only group endorsed HCl, buffered, nitrate, multi-ingredient blend, or ‘not sure’ without endorsing monohydrate or micronized.

The overall median age was 55 years (IQR: 46–63; Table 1), with women slightly older than men (56 [47–63] vs. 54 [44–63]; *p* = 0.007). Median BMI was 26 kg/m^2^ overall; men had a higher median BMI than women (27 [25–31] vs. 25 [22–30]; *p* < 0.001). The sample was predominantly middle-aged to older (23% aged 40–49; 29% aged 50–59; 27% aged 60–69), with 13.1% under age 39. Most were moderately or highly active (43% and 45%, respectively). Activity level did not differ by gender (*p* = 0.5).

Bivariate gender comparisons identified significant differences across almost all outcomes after BH-FDR correction. The largest effects were for duration of use (rank-biserial |r| = 0.307), knowledge confidence (Cramér’s V = 0.266), and knowledge score (|r| = 0.228). Overall, 55% perceived benefit, 98% intended to continue, and mean satisfaction was 7.8/10.

### 3.2. Supplementation patterns

Creatine use patterns were examined by gender identity (Table 2) and by age group (Table S1).

Duration of use differed by gender (*p* < 0.001): 28% of men reported ≥3 years of use compared with 7% of women, whereas women were more likely to report 1–3 yr of use (32% vs. 27%). Cycling behaviour was reported by 3.9% overall and was more common in men than women (5.6% vs. 2.0%).

The median daily dose was 5.00 g/day (IQR: 5.00–5.00 among women, 5.00–10.00 among men; Wilcoxon *p* < 0.001). Men reported a higher mean dose than women (7.50 ± 3.64 vs. 5.93 ± 2.42 g/day).

Loading phase use was more common among men (20% vs. 12%; *p* < 0.001), while more women reported being unsure what a loading phase is (13% vs. 6%).

Once-daily dosing predominated in both genders (84% of women; 75% of men), while multiple-daily dosing was more common among men than women (18% vs. 7.5%).

Patterns were broadly similar across age groups for loading phase use (*p* = 0.2), dose category (*p* = 0.8), and dosing frequency (*p* = 0.2), though duration and daily dose varied significantly (*p* < 0.001 and *p* = 0.016).

### 3.3. Goals for use, perceived benefits, and expectation-experience gaps

In adjusted logistic regression (Table 3; *N* = 2,057), older age and female gender identity were associated with lower odds of perceived benefit (age OR/year = 0.987, 95% CI: 0.979–0.994; female OR = 0.66, 95% CI: 0.55–0.80; both *p* < 0.001). Duration (OR/category = 1.20, 95% CI: 1.11–1.29; *p* < 0.001), activity (OR = 1.21, 95% CI: 1.07–1.38; *p* = 0.003), and knowledgeθ (OR = 1.299, 95% CI: 1.145–1.475; *p* < 0.001) were positively associated with benefit. Daily dose (per 5 g) and co-supplements were not significant.

**Table 3.** Multivariate predictors of perceived benefit.

Characteristic	OR	95% CI	*p*-value
Age	0.987	0.979, 0.994	**<0.001**
Gender (women)	0.662	0.550, 0.797	**<0.001**
Dose (per 5g)	1.002	0.995, 1.011	0.6
Duration (ordinal)	1.198	1.109, 1.294	**<0.001**
Activity (ordinal)	1.211	1.066, 1.375	**0.003**
Knowledge (IRT theta)	1.299	1.145, 1.475	**<0.001**
*N* co-supplements	1.027	0.973, 1.083	0.3

Abbreviations: CI = Confidence Interval, OR = Odds Ratio. Note: Side-effect history excluded (contemporaneous co-outcome; conditioning introduces collider bias). N goals excluded (mechanically coupled with outcome: endorsing more goals increases probability of perceiving at least one benefit). Dose: per 5-g increment. Knowledge: IRT theta (EAP scores from GPCM; mean approximately 0, SD approximately 1).

In the gender × dose interaction model (*N* = 2,057), daily dose was positively associated with perceived benefit in men (dose OR/5 g = 1.53, 95% CI: 1.25–1.87; *p* < 0.001), with a significantly attenuated association among women (woman × dose OR = 0.65, 95% CI: 0.53–0.80; *p* < 0.001).

**Figure 2.**
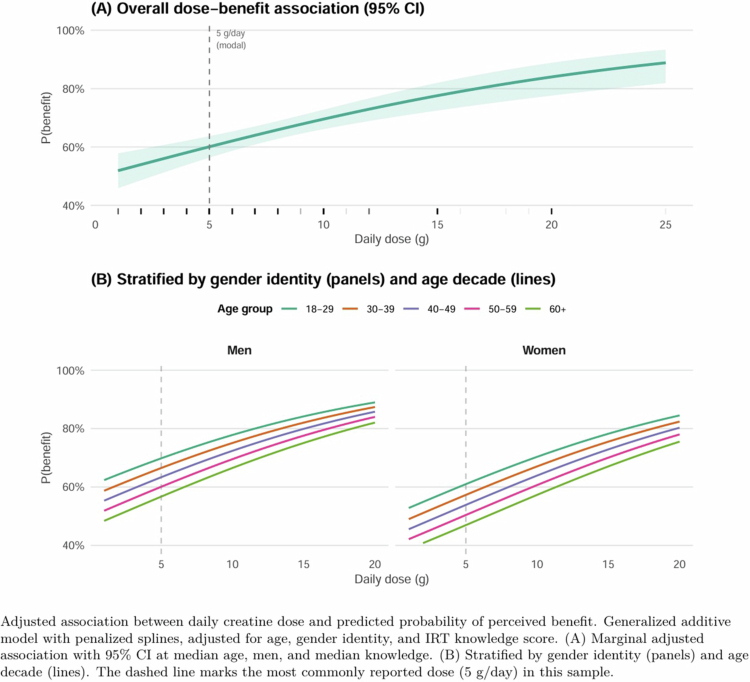
Adjusted association between creatine dose and perceived benefit. (A) Marginal adjusted association between daily creatine dose and perceived benefit with 95% confidence interval, derived from a generalised additive model with penalised splines, adjusted for age, gender identity, and IRT knowledge *θ* (evaluated at median age, men, and median knowledge). (B) Stratified by gender (panels) and age decade (lines). Dashed line indicates the most commonly reported dose (5 g/day) in the sample.

A GAM characterising the adjusted association between dose and perceived benefit (Figure 2) showed that predicted perceived benefit increased with daily dose after adjustment for age, gender, and knowledge *θ*. Predicted benefit remained higher among men than women across the dose range and decreased with increasing age.

**Figure 3.**
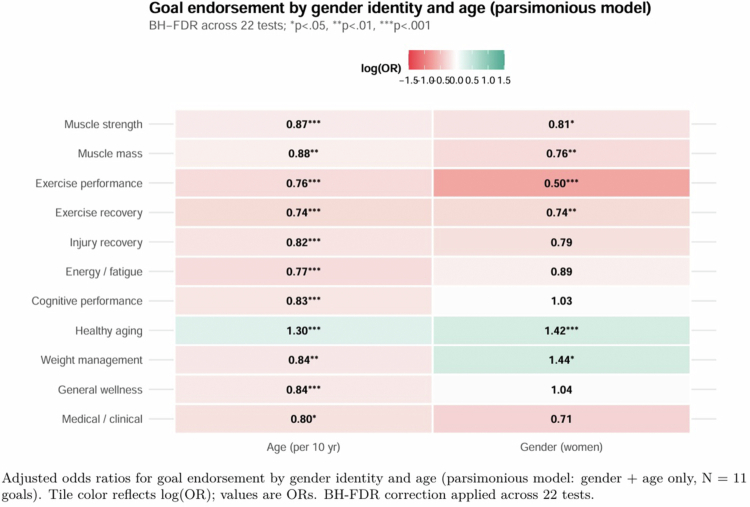
Age and gender identity predictors of creatine supplementation goal endorsement. Odds ratios from parsimonious logistic regression models with age (per 10-year increase) and gender identity (woman vs. man) as predictors, fit separately for each of 11 goals. BH-FDR correction applied across 22 tests. *p < .05, **p < .01, ***p < .001. OR < 1 (red shading) indicates lower endorsement; OR > 1 (green shading) indicates higher endorsement.

Goal for creatine use differed by gender identity and age (Figure 3; Figure S1). Cognitive performance was the most endorsed goal overall (67.8% of respondents), followed by healthy aging and general wellness. Women were less likely than men to endorse performance-oriented goals (exercise performance OR = 0.50; muscle mass OR = 0.76; recovery OR = 0.74; strength OR = 0.81; all p ≤ 0.022) and more likely to endorse healthy aging (OR = 1.42, *p* < 0.001). Age was inversely associated with most goals (OR/10 years = 0.73–0.88), except healthy aging, which increased with age (OR/10 years = 1.30, *p* < 0.001).

Within-person paired analyses comparing goal endorsement with the matched perceived-benefit item (Table 4) showed that, across the five domains where a matched benefit item was available, the conditional proportion of goal-endorsers who reported the matched perceived benefit ranged from 35.6% (cognitive performance) to 45.3% (muscle strength). The corresponding unmet-expectation rate (1 minus the conditional benefit) ranged from 54.7% (strength) to 64.4% (cognitive performance). All five paired comparisons rejected the null of equal discordance (McNemar exact-binomial *p* < .001), with substantially more endorser-without-benefit pairs than benefit-without-endorser pairs in every domain. The largest absolute discordance was for cognitive performance.

**Table 4.** Goal endorsement, conditional perceived benefit, and expectation gap.

Goal domain	Endorsed (%)	*N* endorsers	Benefit among endorsers (%)	Unmet (%)	Endorsed without benefit		Benefit without endorsing	McNemar p
Cognitive performance	67.8%	1476	35.6%	64.4%	950	73		<.001
Energy/fatigue	37%	805	37.5%	62.5%	503	189		<.001
Exercise recovery	45.7%	995	39.8%	60.2%	599	135		<.001
Muscle mass	48.1%	1048	41.2%	58.8%	616	153		<.001
Muscle strength	58.9%	1282	45.3%	54.7%	701	157		<.001

Note: Benefit among endorsers = conditional proportion: of those who endorsed the goal, what percentage reported the matched perceived benefit. Unmet = 1 minus conditional benefit rate. Endorsed without benefit and Benefit without endorsing are the McNemar discordant counts. McNemar p tests the null that these discordant counts are equal (exact binomial test). All five comparisons remain significant under Bonferroni-Holm correction across the paired-test family (adjusted p < .001 each).

In adjusted benefit-domain models (Figure S2), female gender identity was associated with lower odds of benefit endorsement for strength, recovery, cognition, energy, and mood, while knowledge *θ* was positively associated across all domains. Activity predicted benefit for strength, muscle mass, cognition, and mood; duration was associated with strength and muscle mass benefit.

### 3.4. Creatine knowledge and gender differences in response uncertainty

**Figure 4.**
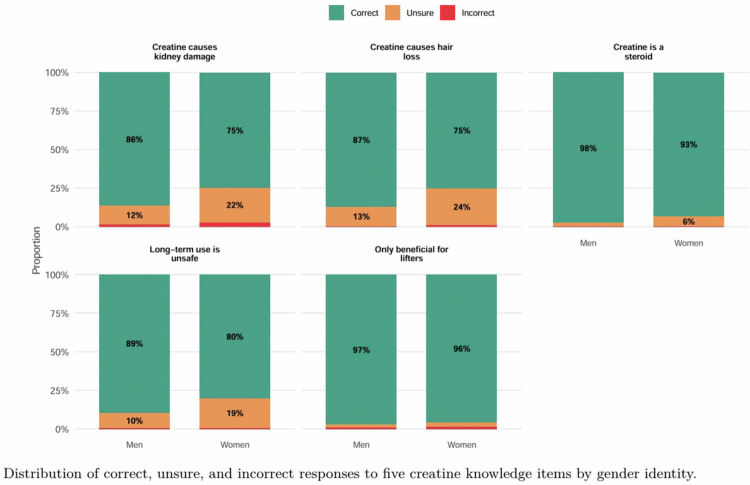
Accuracy of creatine knowledge items by gender identity. Stacked bar charts showing response distributions (correct, unsure, incorrect) for six knowledge statements, stratified by gender identity (man/woman). Items are organised by IRT 2PL discrimination (top row: high discrimination; bottom row: low to moderate discrimination).

Correct responding was highest for “creatine is not only for weightlifters” (96.6%) and “creatine is not a steroid” (95.5%). For kidney damage and hair loss, most respondents answered correctly (80.6% and 81.2%, respectively), with high rates of “unsure” responding (~17–18%; Figure 4).

Gender differences in response patterns were pronounced (Table S3; Figure 4). Women selected “unsure” more frequently than men across five of six items, while men were more likely to choose definitive responses. Gender differences were significant for five of six items by Fisher’s exact tests (all *p* < 0.001), with no difference for the “only beneficial for lifters” item (*p* = 0.319). The strongest differences were observed among participants aged ≥50 years.

**Figure 5.**
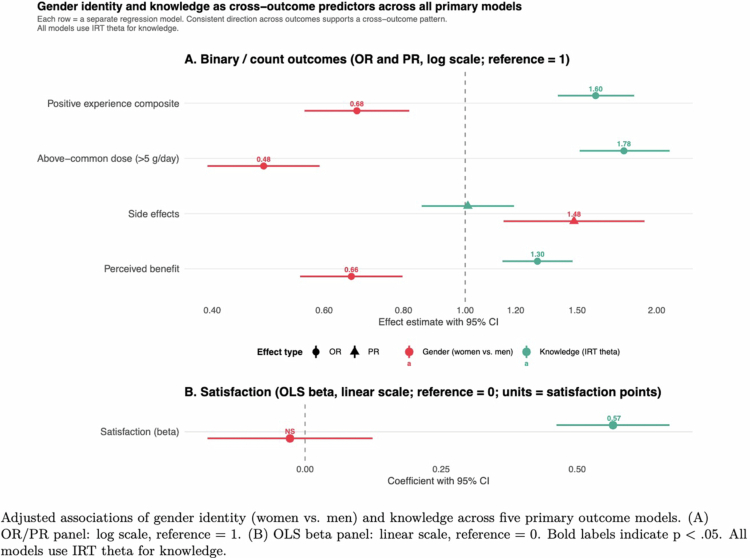
Associations of gender identity and knowledge with creatine supplementation outcomes. (A) Binary and count outcomes (OR and PR on log scale; reference = 1). (B) Satisfaction (OLS beta on linear scale; reference = 0; units = satisfaction points). Each row represents a separate regression model. Consistent direction across outcomes supports gender identity and knowledge as cross-outcome correlates. All models use IRT *θ* for knowledge.

IRT 2PL parameters for the five-item battery (Supplementary Table S2) showed high discrimination for kidney damage (a = 4.83) and hair loss (a = 2.93), moderate discrimination for long-term safety (a = 1.95) and steroid categorisation (a = 1.77), and low discrimination for the “only beneficial for lifters” item (a = 1.17). Female gender identity and knowledge were consistent predictors across all primary outcomes (Figure 5).

### 3.5. Side effects

**Figure 6.**
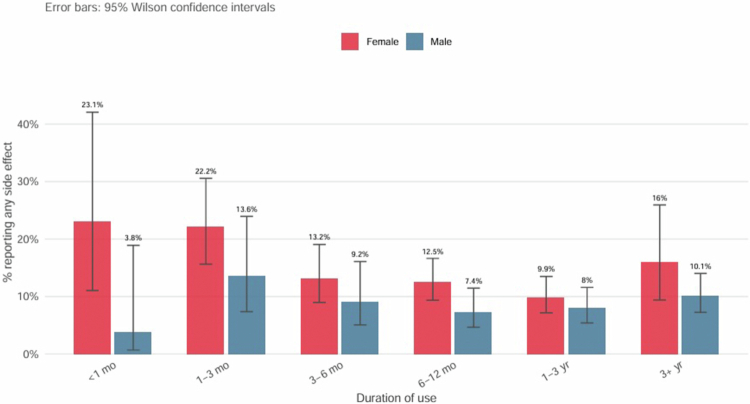
Prevalence of reported creatine side effects by duration of use. Percentage reporting any self-reported side effect at each duration category, stratified by gender identity. Error bars represent 95% Wilson confidence intervals.

Self-reported side-effect prevalence by gender identity and duration of use is shown in Figure 6. Across durations, approximately 10–15% reported any side effect at a given timepoint, with a higher proportion of women than men across all durations. The highest prevalence was observed among newer female users (23.1% at <1 month). A secondary increase was observed among women reporting ≥ 3 years of use (16.0%).

In adjusted modified Poisson models (Table 5), female gender identity was associated with higher prevalence of self-reported side effects (PR = 1.48, 95% CI 1.15–1.91; *p* = 0.002). No other covariates were significantly associated at *α* = 0.05, including duration of use (PR = 0.91, 95% CI: 0.82–1.01; *p* = 0.088).

**Table 5.** Predictors of side-effect reporting.

Covariate	PR	95% CI	*p*-value
Age (years)	0.99	0.98—1.00	0.231
Gender (women)	1.48	1.15—1.91	**0.002**
Dose (per 5g)	1.00	1.00—1.01	0.480
Duration (ordinal)	0.91	0.82—1.01	0.088
Activity (ordinal)	0.97	0.81—1.15	0.701
Knowledge (IRT theta)	1.01	0.85—1.19	0.911
Used loading phase	1.10	0.79—1.54	0.578
*N* co-supplements	1.03	0.96—1.10	0.440

Note: CI = confidence interval; PR = prevalence ratio. Robust sandwich standard errors (HC0) used to account for Poisson overdispersion.

For self-reported side-effect types (Figure S4), bloating and water retention were most commonly reported and showed the clearest gender and age gradients (bloating: 18.2% in women aged 30–39 vs. 1.4–2.4% in men across most age groups; water retention: 13.6% in women aged <30 declining to 3.6% by age 60 + , compared with 1.4–3.2% in men); muscle cramps were uncommon (<2%).

### 3.6. Satisfaction and loyalty

Overall satisfaction averaged 7.8/10, highest among men aged 50+ (mean 8.3) and lowest among women aged < 50 (mean 7.5). Intent to continue was reported by 98% and did not differ by gender or age; because intent-to-continue is endorsed by nearly the entire sample, it contributes minimal discriminative information to the positive experience composite, which is therefore effectively determined by perceived benefit and satisfaction ≥8.

In the primary OLS model excluding perceived benefit (Table 6; *N* = 2,057; R^2^ = 0.176), satisfaction was higher with greater knowledgeθ (*β* = 0.57, *p* < 0.001), longer duration (*β* = 0.28, *p* < 0.001), higher activity (*β* = 0.13, *p* = 0.014), and more goals endorsed (*β* = 0.09, *p* < 0.001), and lower with self-reported side-effect history (*β* = –0.39, *p* = 0.001). Gender identity and dose were not independently associated. In the secondary model including perceived benefit (R^2^ = 0.364), perceived benefit was strongly associated with satisfaction (Benefit: Yes *β* = 2.5, *p* < 0.001; Benefit: Unsure *β* = 1.1, *p* < 0.001).

**Table 6.** Predictors of creatine supplementation satisfaction.

Characteristic	Beta	95% CI	*p*-value
Age	-0.01	-0.02, 0.00	**0.002**
Gender (women)	-0.03	-0.18, 0.12	0.7
Dose (per 5g)	0.00	0.00, 0.01	0.14
Duration	0.28	0.22, 0.34	**<0.001**
Activity	0.13	0.03, 0.23	**0.014**
Knowledge (IRT theta)	0.57	0.46, 0.67	**<0.001**
Side-effect history	-0.39	-0.63, -0.16	**0.001**
Co-supplements	0.04	0.00, 0.08	0.062
*N* goals	0.09	0.05, 0.12	**<0.001**

Abbreviation: CI = Confidence Interval. Note: Benefit perception excluded as endogenous predictor (shares measurement error with satisfaction). N = 2058, R² = 0.176. OLS estimated on 1–10 satisfaction scale.

**Figure 7.**
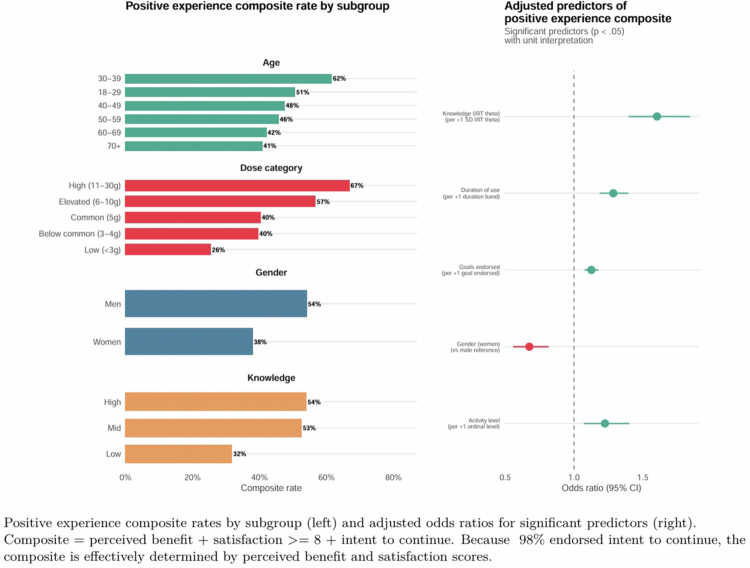
Predictors and characteristics of the positive experience composite. Left panel: positive experience composite rates (perceived benefit + satisfaction ≥8 + intent to continue) by age group, dose category, knowledge tertile, and gender identity. Right panel: adjusted odds ratios with 95% confidence intervals from logistic regression. Significant predictors (*p* < .05) shown with unit interpretation. Because ~98% of the sample endorsed intent to continue, the composite is effectively determined by perceived benefit and satisfaction ≥ 8.

The positive experience composite rate was 54% in men and 38% in women (Figure 7). Composite rates peaked at 62% (ages 30–39), declining to 41% (70+), with a dose gradient: 26% among those taking <3 g/day, 40% at the modal 5 g/day dose, and 67% among those reporting 11–30 g/day. Rates also increased across knowledge tertiles (low: 32%; mid: 53%; high: 54%).

### 3.7. Above-common dosing behaviour

Overall, 34% reported daily doses >5 g/day (Supplementary Table S4). In adjusted logistic regression (*N* = 2,050), above-common dosing was associated with higher knowledge *θ* (OR = 1.78, *p* < 0.001), more co-supplements (OR = 1.20 per supplement, *p* < 0.001), longer duration (OR = 1.21 per category, *p* < 0.001), prior loading phase use (OR = 1.42, *p* = 0.008), and more goals endorsed (OR = 1.06, *p* = 0.020). Female gender identity was associated with lower odds (OR = 0.48, *p* < 0.001), and age showed a small positive association (OR = 1.01/year, *p* = 0.021). Among men, 10 g/day or more was reported by 18–44% across age groups, peaking at ages 40–49 (44.4%) and 50–59 (43.7%); above-common use was lower among women (range 4.3–18.8%).

### 3.8. Free-text responses

Of 2,233 respondents, 1,501 provided valid free-text responses. The most frequent themes were cognitive/brain benefits (14.8%; *n* = 222), wishing they had started sooner (12.3%; *n* = 184), and dosing guidance (9.7%; *n* = 146).

By domain, Benefits Awareness was most common (300 mentions), followed by Protocol Guidance (246), Wish Started Sooner (184), and Myth Correction (181).

Age and gender patterning showed that cognitive/brain themes were prominent across groups, with higher endorsement among men aged 50–59 and women aged 30–39. “Wish started sooner” was more common among older participants, and dosing-guidance themes were more common among women and among those aged 40–59.

### 3.9. Cross-domain network analysis (exploratory)

The MGM produced an exploratory network of 35 nodes with conditional associations across all survey domains. The strongest cross-domain edges (Supplementary Table S5; Figure S5) included: bloating and female gender identity (0.273); cognition goal endorsement and cognitive-benefit free-text themes (0.253); long-term safety knowledge and intent to continue use (0.243); and female gender identity with shorter duration (0.208) and lower dose (0.202). Bootstrap edge stability is reported in Supplementary Table S7; two of these edges (bloating–women, only-lifters–cognition) had bootstrap inclusion below 65% and are flagged as Unstable, and we therefore do not interpret them as reliable individual associations. The long-term safety—intent-to-continue edge fell in the Moderate stability tier (76% inclusion) and is interpreted only at the directional level. Centrality ordering was stable on case-dropping bootstrap (CS-coefficient = 0.7; Supplementary Figure S6).

Centrality analyses highlighted bloating among side-effect nodes as highly connected. Among knowledge items, kidney damage and long-term safety were most central. Bridge centrality identified female gender identity and satisfaction as prominent cross-community connectors.

In an exploratory IRT-by-network analysis (now reported in Supplementary Figure S7), kidney damage showed the highest IRT discrimination (a = 4.83) with above-median network centrality, identifying it as the strongest candidate keystone misconception in this sample. Hair loss showed moderately high discrimination (a = 2.93) with centrality just above the median. Long-term safety showed high network centrality with moderate discrimination (a = 1.95).

## 4. Discussion

To our knowledge, this is one of the largest cross-sectional surveys of real-world creatine supplementation practices, knowledge, and self-reported outcomes in a free-living, community-based U.S. population. The median respondent was 55 years old, reported 5 g/day, and was motivated by healthy aging and cognition rather than muscle or strength goals. Despite 98% intending to continue use and a mean satisfaction rating of 7.8 out of 10, only 55% perceived benefit from creatine supplementation. Within-person paired comparisons of goal endorsement and matched perceived benefit showed an excess of unmet expectations across every measured domain, with the largest unmet rate for cognitive performance (64.4% of cognitive-goal endorsers did not perceive a matched cognitive benefit) and the smallest for muscle strength (54.7%), mirroring the differential strength and consistency of the underlying evidence base [2,18]. Women and older adults reported lower knowledge confidence, lower perceived benefit, and lower self-reported satisfaction than younger men, despite representing populations for whom the underlying clinical evidence may be most relevant. We interpret these findings descriptively, given the cross-sectional design and the reliance on self-report.

The demographic profile differs from those traditionally enroled in supplementation trials. Healthy aging was the only goal that increased in endorsement across successive age groups, and “wishing I had started creatine sooner” was the second most common free-text theme (12.3%; *n* = 184), reported more often by older participants. In adjusted models, older age was associated with lower odds of perceived benefit (OR per year 0.987; 95% CI 0.979–0.994), even among those reporting consistent daily use. The age gradient in healthy-aging and cognitive-performance goal endorsement is consistent with a maintenance motivation rather than an enhancement motivation in older respondents, who also reported a high prevalence of co-supplementation with vitamin D, magnesium, omega-3, and multivitamins; the network analysis correspondingly couples age with healthy-aging goal endorsement (edge weight = 0.216; bootstrap-stable). These patterns are also compatible with a lifespan-oriented framing in which adults initiate creatine relatively late in life. Progressive sarcopenia and bone loss are detectable from the fourth decade [37], and age-related reductions in skeletal muscle phosphocreatine and brain creatine concentrations follow a similar trajectory [38,39]. These findings underscore the importance of earlier, lifespan-oriented education rather than messaging that begins after functional decline is apparent.

The disconnect between cognitive goal endorsement and perceived cognitive benefit deserves particular attention. Meta-analytic evidence supports small to moderate effects on memory and processing speed, with largest benefits in older adults, during sleep deprivation, and in populations with low dietary creatine intake such as vegetarians [18,19]. The cognitive expectation gap in our sample may be partially interpreted as a dose-translation issue: the most commonly reported dose in our sample (5 g/day) is at the low end of the cognitive-trial dose range (typically 5–20 g/day). This pattern is consistent with the cognitive trial literature, which reports the largest effects at higher doses and under conditions of energetic demand or lower baseline creatine availability [19]. Older adults could theoretically benefit, given age-related changes in brain creatine and phosphocreatine concentrations, yet this population also reported the lowest perceived cognitive benefit rates in this sample [13,16]. This suggests that public-facing guidance for cognitive applications may benefit from indicating that effects may require doses above the modal 5 g/day and a defined time course, with research still emerging [40]. Consistent with this gap, cognitive and brain health was the most endorsed information need in free-text responses (14.8%; *n* = 222). Clearer guidance on the conditions and timescales for cognitive benefit could better calibrate expectations and reduce premature discontinuation.

Women in this sample consistently reported a less favourable supplementation experience than men: lower knowledge confidence, higher uncertainty across knowledge items, higher self-reported side-effect prevalence, lower perceived benefit, and a lower positive experience composite rate (38% vs. 54%). In adjusted models, female gender identity was associated with higher self-reported side-effect prevalence (PR = 1.48; 95% CI 1.15–1.91) and lower odds of perceived benefit (OR = 0.66; 95% CI 0.55–0.80) independent of measured covariates. The women in our sample reported a modal dose of 5 g/day, shorter durations of use, more knowledge uncertainty, and higher likelihood of being unsure about a loading phase. These patterns suggest that the gender gap in supplementation experience in our sample may reflect differences in dose, duration, and access to information. Reviews of women’s-health applications indicate creatine remains efficacious across the female lifespan when dosed appropriately, and pooled trial and surveillance evidence does not show an excess of adverse events in women relative to men or to placebo [20,21].

Behavioural patterns are consistent with this framing. A significant gender-by-dose interaction (woman × dose OR = 0.65; 95% CI 0.53–0.80; *p* < 0.001) showed that the positive association between dose and perceived benefit observed in men was attenuated in women, although the women in our sample also reported lower average doses and shorter durations of use than men, with only 7% reporting three or more years compared to 28% of men. Duration was among the strongest predictors of both perceived benefit and satisfaction in adjusted models, consistent with the time required for skeletal muscle creatine saturation and the longer windows over which cognitive and systemic effects emerge [18,41]. Self-reported side-effect prevalence was highest among women in the first three months of use (23.1% at <1 month; 22.2% at 1–3 months), and self-reported side-effect history was the strongest negative correlate of satisfaction (*β* = −0.39). Early reported side effects and shorter duration of use co-occur in our cross-sectional data; the directionality of this relationship cannot be determined from these data, but is consistent with a pattern in which early reported tolerability concerns coincide with discontinuation before benefits accumulate. Pooled trial and surveillance data find no excess of adverse events in women relative to men or to placebo, suggesting that the higher self-reported prevalence in our sample is unlikely to reflect a women-specific physiological adverse-event signal [6,24].

Knowledge uncertainty may also influence these patterns. The female pattern was characterised by elevated uncertainty rather than active endorsement of misinformation, with women selecting “unsure” more often than men on four of five items, particularly those relating to tolerability and body-composition effects. This uncertainty may reflect limited access to clear, gender-relevant guidance and is better addressed through proactive, evidence-based education than through myth correction alone. Public-facing communication about creatine and hydration should distinguish the well-documented transient intracellular fluid shift during the first ~5–7 days of loading from the colloquial misconception of long-term whole-body water retention or bloating, which is not supported by trial evidence [6,42].

As an exploratory exercise, we integrated IRT discrimination with cross-domain network centrality to identify candidate keystone misconceptions warranting prospective evaluation (Supplementary Figure S7). The kidney damage item most clearly met both criteria, with the highest IRT discrimination (a = 4.83) and above-median network centrality, consistent with kidney damage functioning as a persistent misconception that, in this sample, separates more- from less-knowledgeable respondents efficiently. The kidney damage statement is not supported by the trial or surveillance literature: pooled trial and adverse-event surveillance analyses find no excess of renal adverse events with creatine relative to placebo, a recent meta-analysis found that creatine has no significant effect on glomerular filtration rate (how well kidneys filter waste), and Mendelian randomisation analyses do not support a causal effect of elevated serum creatine concentrations on renal function [6,31,43]. The persistence of these misconceptions is consistent with broader observations that non-professional information channels, including social media and product marketing, may perpetuate misinformation [32].

The long-term safety item showed discrimination at the median and a moderately stable conditional association with intent to continue (edge weight = 0.243; 76% bootstrap inclusion, Moderate stability tier), consistent with the hypothesis that long-term safety beliefs track behavioural persistence; directionality cannot be determined from cross-sectional data. The keystone-misconception framework is exploratory and hypothesis-generating, requiring replication with larger item batteries and prospective designs before informing intervention priorities. The network also reflected the cognitive reorientation of the sample: cognition goal endorsement and cognitive-benefit free-text themes appeared as a connected pair, and nootropics clustered with creatine, consistent with survey data identifying creatine as the second most commonly used nootropic among physically active individuals [44]. These findings collectively suggest that the creatine experience in this population is embedded in a broader, cognitively oriented supplementation practice.

Across analyses, knowledge was the most consistent positive correlate of satisfaction (*β* = 0.57 per SD of IRT theta; *p* < 0.001), outperforming dose, duration, and activity. Female gender identity was not independently associated with lower satisfaction in the primary model (*β* = –0.03, *p* = 0.7), and when perceived benefit and self-reported side-effect history were added the gender coefficient became positive (*β* = 0.15, *p* = 0.027), indicating that women who perceive benefit and tolerate supplementation report satisfaction at least equivalent to their male counterparts. The gender gap in satisfaction therefore appears attributable to differences in perceived benefit and self-reported side-effect burden rather than any direct association of gender with the satisfaction rating itself.

Taken together, these patterns generate three hypotheses warranting prospective evaluation. First, addressing persistent misconceptions about kidney safety and long-term tolerability may be useful, given that the trial and surveillance literature does not show a creatine-specific adverse-event signal and that long-term-safety beliefs were associated with intent to continue in our cross-sectional network. Second, structured onboarding guidance that frames the only well-documented early hydration effect as a transient, intracellular shift during loading, rather than as long-term fluid retention or bloating, may help calibrate expectations among new users, particularly women, who reported the highest rates of self-reported tolerability concerns and the shortest durations of use [42]. Third, benefit expectations, particularly for cognition, could be more tightly calibrated to the dose and time course used in trials. Each of these hypotheses would benefit from prospective educational and dose-finding studies; we do not interpret our cross-sectional findings as direct evidence for intervention efficacy.

## 5. Limitations

Several limitations warrant emphasis. First, the sample was drawn from users of a supplement-tracking application, introducing self-selection toward more engaged consumers; participation also depended on receiving and acting on a digital invitation, so the analysable sample should not be interpreted as representative of all U.S. creatine users. Second, the cross-sectional design, with no creatine-naive comparator, precludes causal inference. Survivorship bias is a particular concern for the duration–satisfaction and knowledge–satisfaction associations: respondents with longer durations, higher doses, and more accurate knowledge are likely to be the subgroup who remained on creatine, which can inflate associations between these predictors and any positive outcome. Third, the survey measured gender identity rather than sex assigned at birth; mechanisms invoking female physiology are inferential, and biological-sex covariate analyses (e.g. menstrual status, menopausal status, hormonal contraception) were not possible. Fourth, the five-item knowledge battery showed modest marginal reliability (0.475); IRT-theta scores are best interpreted at the group level rather than as individual diagnostics. Fifth, side effects were self-reported, retrospective, and unverified; reported prevalence may reflect awareness, prior beliefs, attribution, and co-supplement effects in addition to any physiological signal, and pooled trial and adverse-event surveillance evidence does not show an excess of adverse events on creatine relative to placebo [6,45]. Sixth, the network analysis is exploratory and based on a regularised cross-sectional model; several individual edges are unstable on bootstrap diagnostics (Supplementary Table S7) and should not be interpreted as load-bearing. Finally, asking misconception items in true/false form may itself elicit some of the uncertainty being measured, particularly for items with widespread social-media discourse such as kidney damage and hair loss. Reported knowledge reflects exposure to non-professional information channels, including social media and marketing, which may shape misconception patterns independent of physiological understanding.

## 6. Conclusions

Use patterns, knowledge, and self-reported outcomes differed meaningfully across demographic subgroups in this large community-based sample of current and recent creatine users. Knowledge was the most consistent cross-outcome correlate, associated with perceived benefit, satisfaction, the positive experience composite, and above-common dosing across primary models. Women reported lower satisfaction, higher self-reported side-effect prevalence, and a larger gap between cognitive goals and perceived cognitive benefit, which may reflect an information and behavioural gap. The cognitive performance domain showed the largest within-person unmet-expectation rate among goal-endorsers, despite being the most endorsed reason for use; the cognitive expectation gap may reflect insufficient translation of dose evidence into real-world use rather than absence of efficacy. These findings generate hypotheses for prospective evaluation of education, gender-tailored dosing guidance, and expectation-setting interventions in real-world creatine users.

## Supplementary Material

Supplementary Material SupplementaryFigure1_Burridge.jpg

Supplementary Material SupplementaryFigure2_Burridge

Supplementary Material SupplementaryFigure3_Burridge

Supplementary Material SupplementaryFigure4_Burridge

Supplementary Material SupplementaryFigure5_Burridge

Supplementary Material SupplementaryFigure6_Burridge

Supplementary Material SupplementaryFigure7_Burridge

Supplementary_Tables
