# Supplementary figures and images for: Real-world creatine supplementation: a large-scale cross-sectional study of use, knowledge, and experiences
Source: J Int Soc Sports Nutr. 2026 Jul 21;23(1):2702952. doi: 10.1080/15502783.2026.2702952 (PMC13390171; doi:10.1080/15502783.2026.2702952)

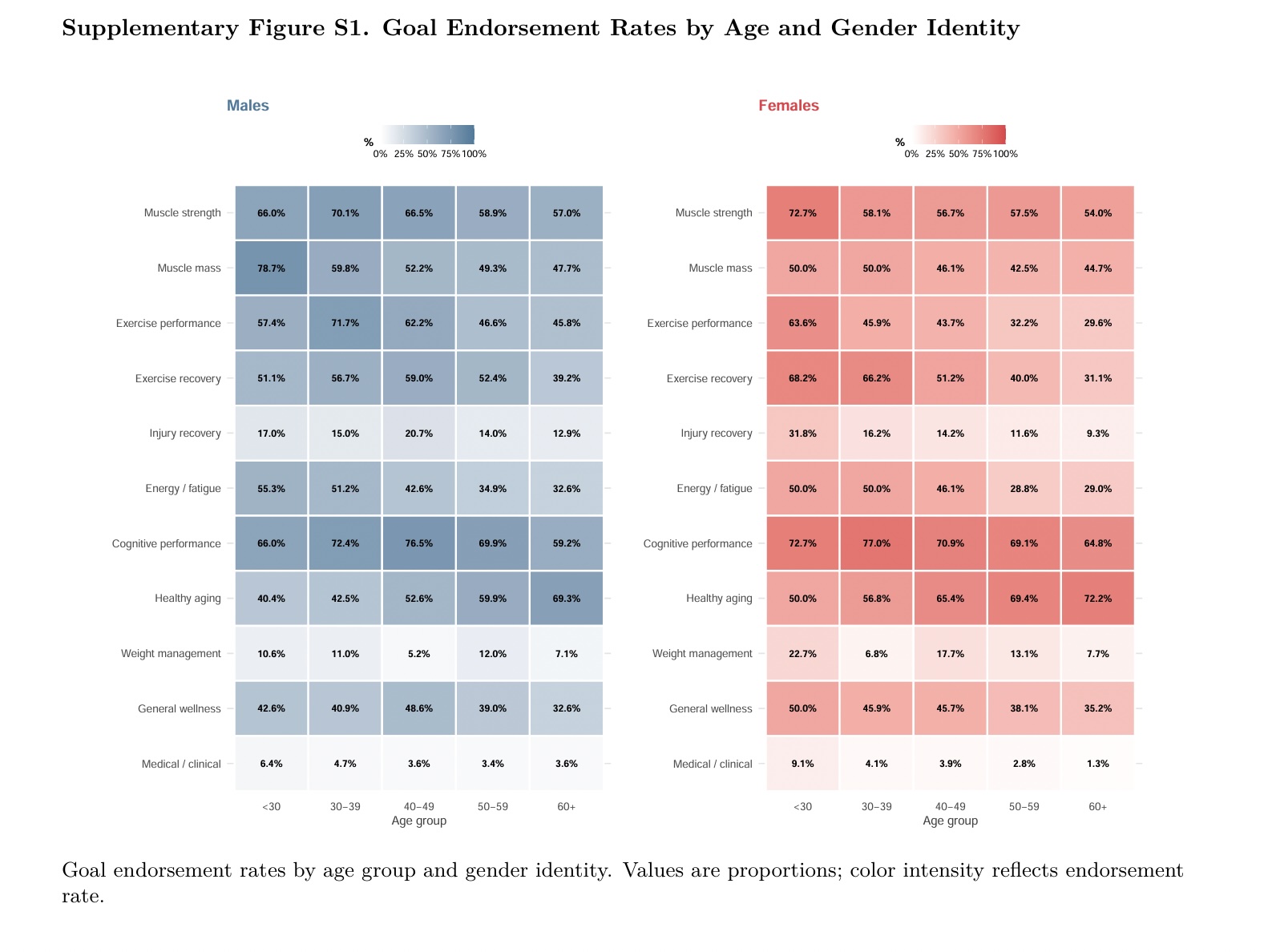

Supplement: Supplementary Material — SupplementaryFigure1_Burridge.jpg [file RSSN_A_2702952_SM0371.jpg]

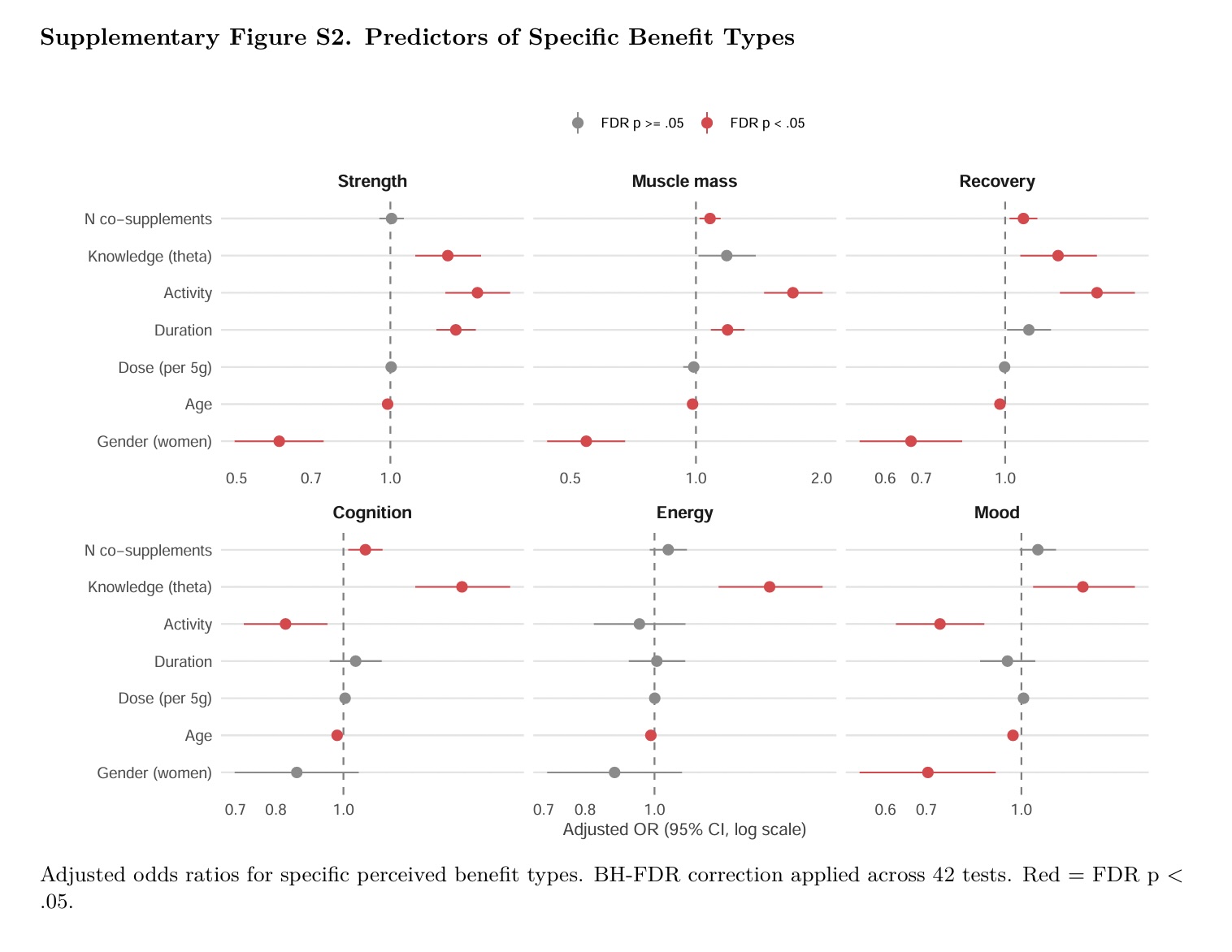

Supplement: Supplementary Material — SupplementaryFigure2_Burridge [file RSSN_A_2702952_SM0376.jpg]

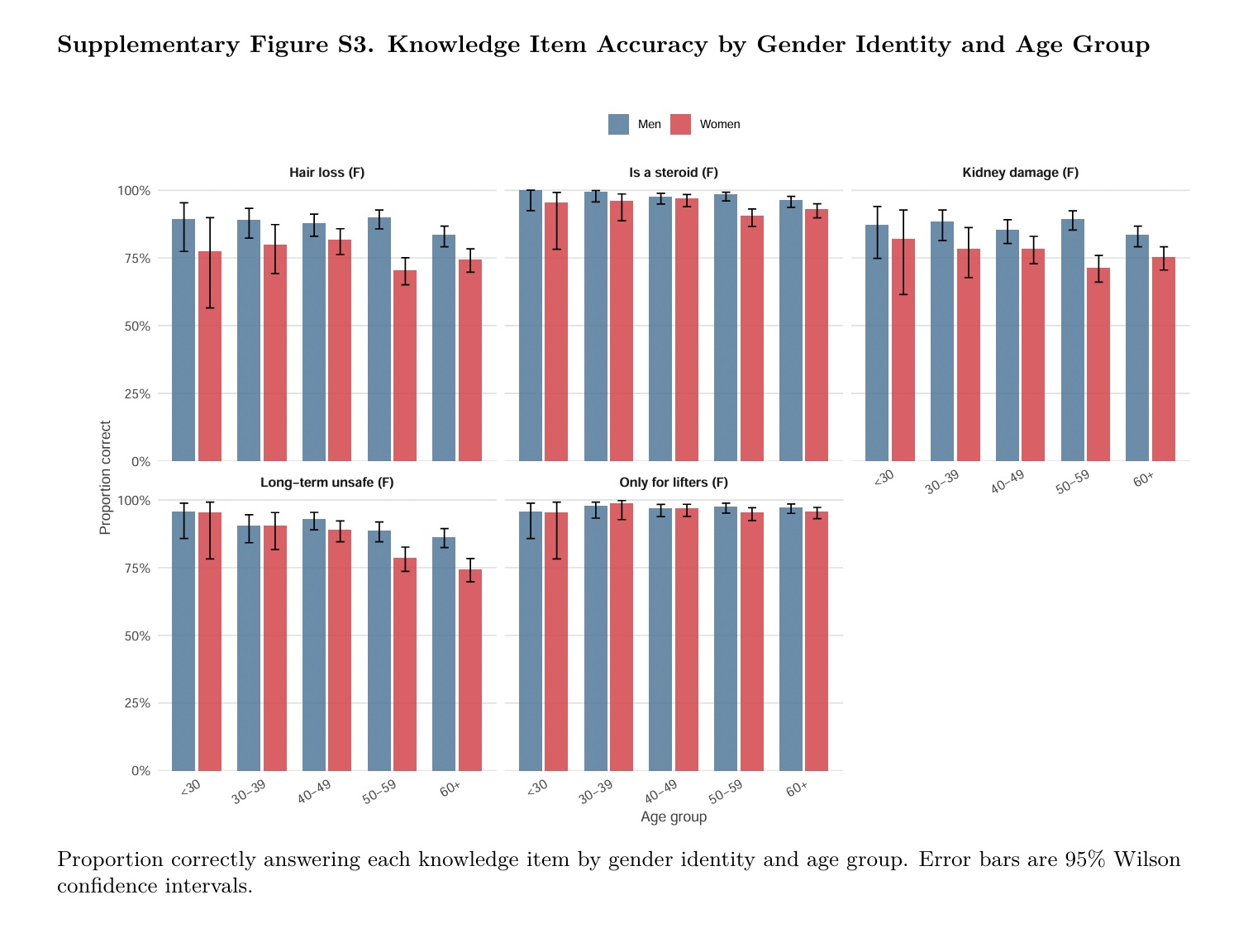

Supplement: Supplementary Material — SupplementaryFigure3_Burridge [file RSSN_A_2702952_SM0492.jpg]

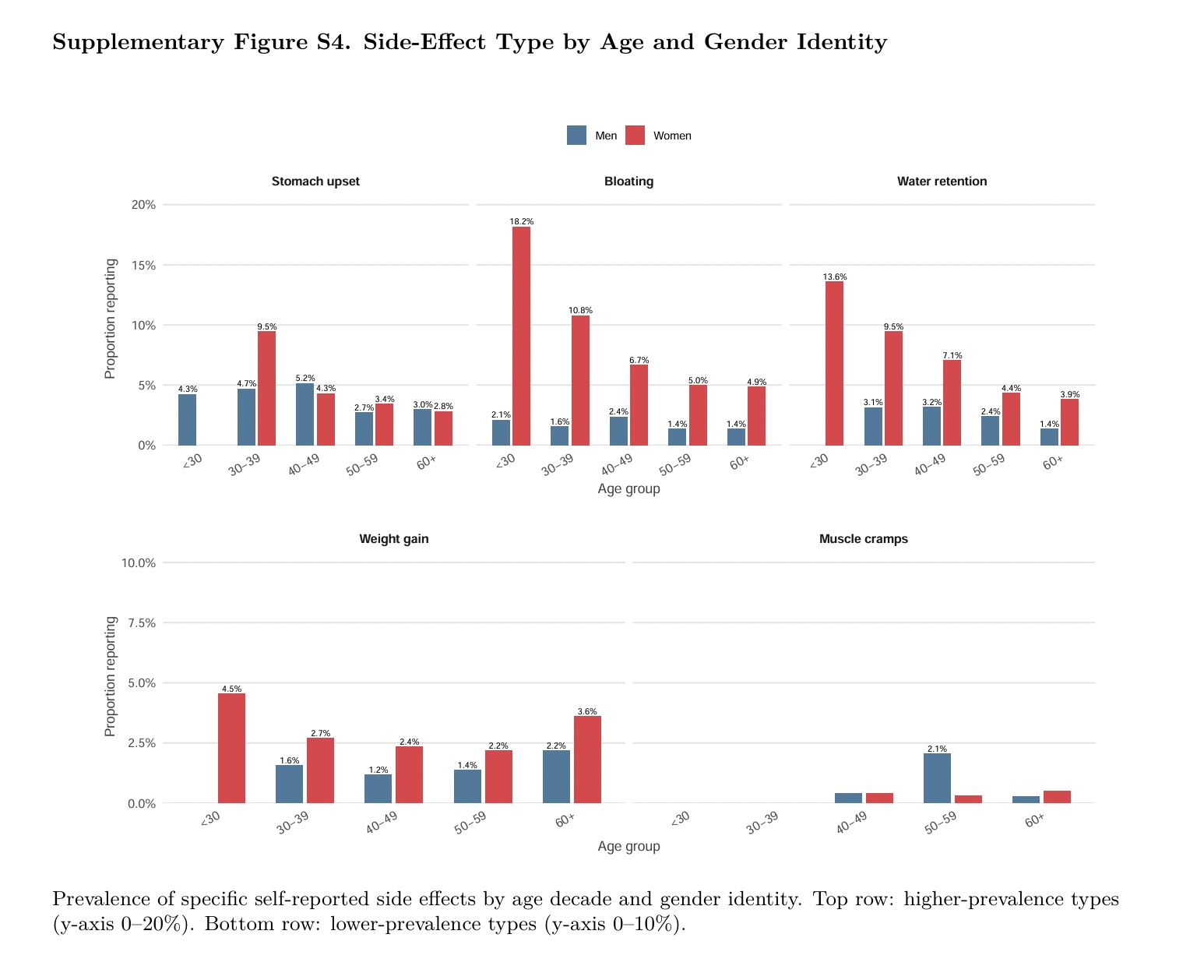

Supplement: Supplementary Material — SupplementaryFigure4_Burridge [file RSSN_A_2702952_SM0503.jpg]

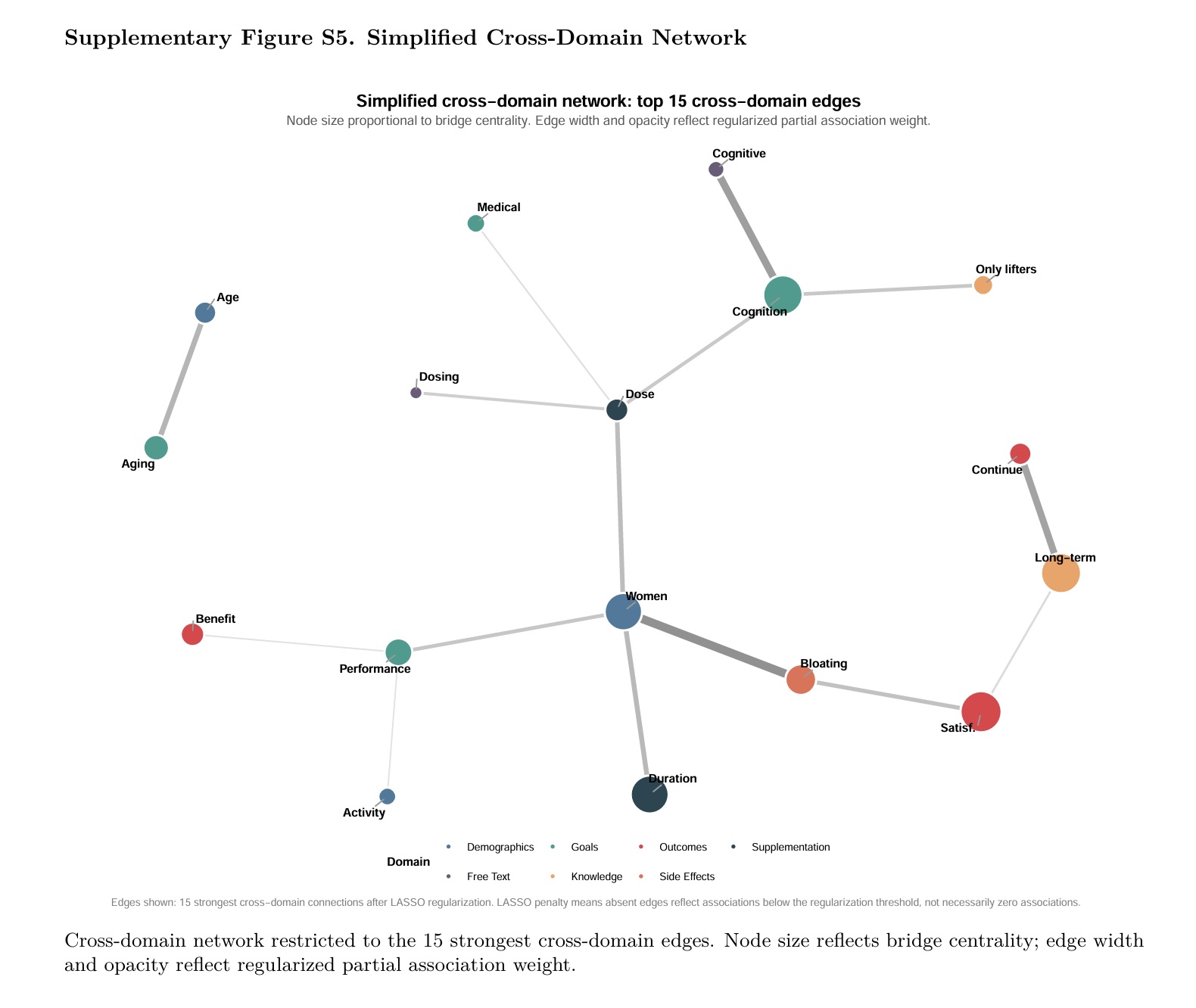

Supplement: Supplementary Material — SupplementaryFigure5_Burridge [file RSSN_A_2702952_SM0506.jpg]

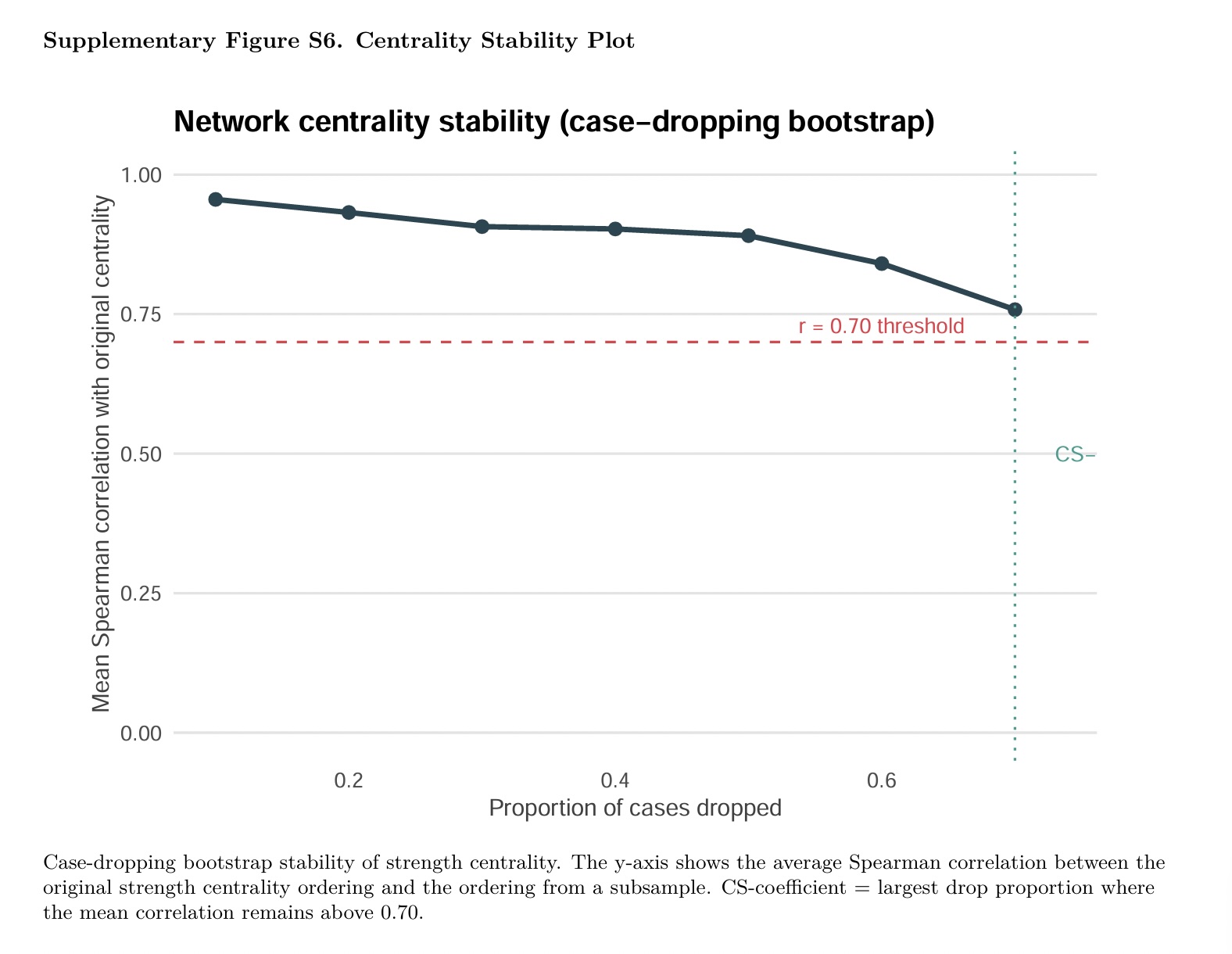

Supplement: Supplementary Material — SupplementaryFigure6_Burridge [file RSSN_A_2702952_SM0537.jpg]

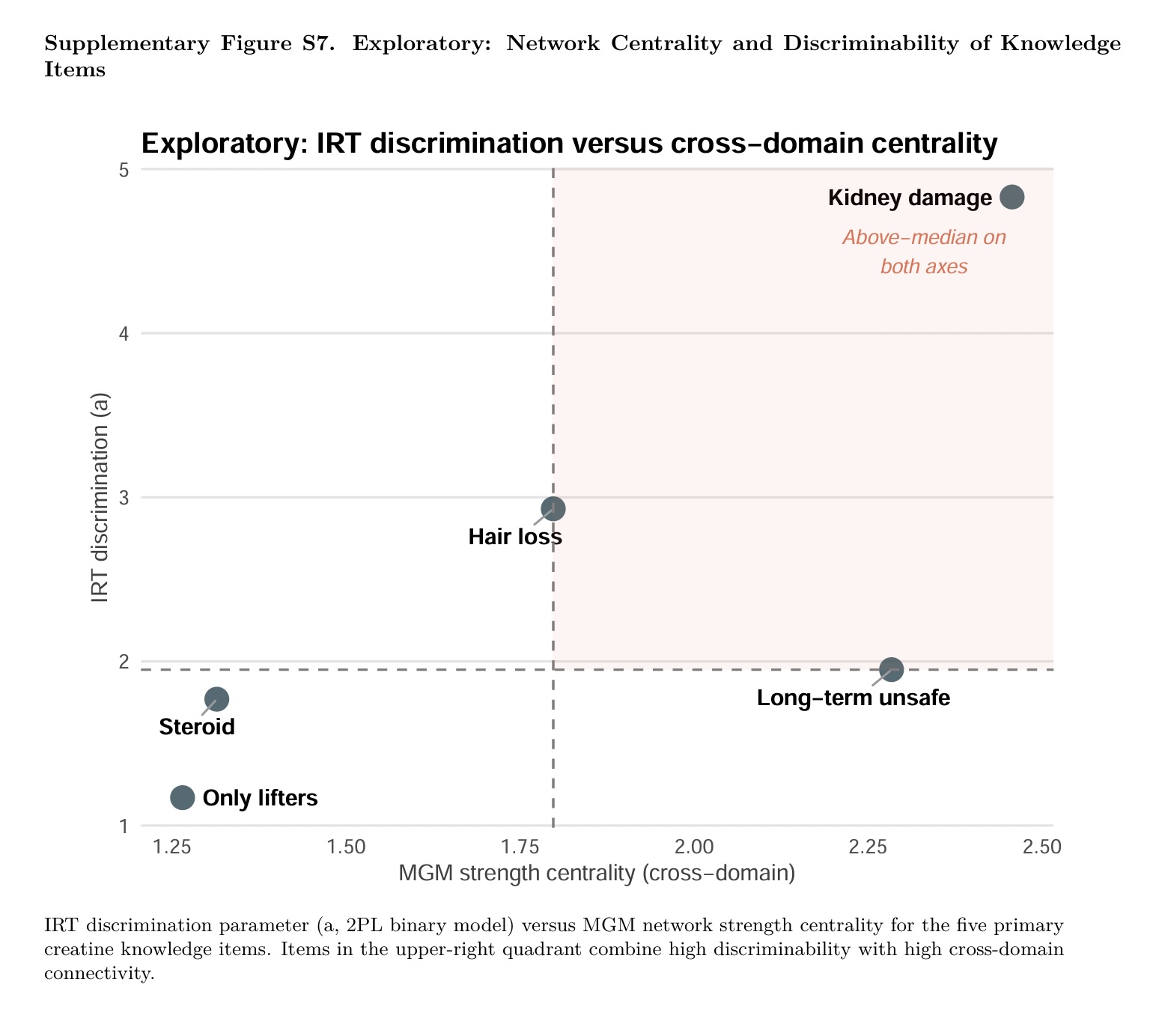

Supplement: Supplementary Material — SupplementaryFigure7_Burridge [file RSSN_A_2702952_SM0557.jpg]
