# Supplementary material for: Real-world creatine supplementation: a large-scale cross-sectional study of use, knowledge, and experiences
Source: J Int Soc Sports Nutr. 2026 Jul 21;23(1):2702952. doi: 10.1080/15502783.2026.2702952 (PMC13390171; doi:10.1080/15502783.2026.2702952)
Supplement: Supplementary_Tables [file RSSN_A_2702952_SM0367.docx]

**Supplementary Table S1.** *Creatine Supplementation Patterns by Age Group*

| **Characteristic** | **Overall**  **N = 2,141** | **18-29**  **N = 69** | **30-39**  **N = 201** | **40-49**  **N = 505** | **50-59**  **N = 612** | **60-69**  **N = 583** | **70+**  **N = 171** | **p-value** |
| --- | --- | --- | --- | --- | --- | --- | --- | --- |
| **Duration of use** |  |  |  |  |  |  |  |  |
| <1 mo | 51 (2.4%) | 2 (2.9%) | 3 (1.5%) | 15 (3.0%) | 10 (1.6%) | 16 (2.7%) | 5 (2.9%) |  |
| 1-3 mo | 180 (8.4%) | 4 (5.8%) | 12 (6.0%) | 37 (7.3%) | 44 (7.2%) | 65 (11%) | 18 (11%) |  |
| 3-6 mo | 277 (13%) | 7 (10%) | 20 (10.0%) | 64 (13%) | 71 (12%) | 87 (15%) | 28 (16%) |  |
| 6-12 mo | 540 (25%) | 11 (16%) | 35 (17%) | 124 (25%) | 159 (26%) | 154 (26%) | 57 (33%) |  |
| 1-3 yr | 631 (29%) | 15 (22%) | 54 (27%) | 141 (28%) | 204 (33%) | 175 (30%) | 42 (25%) |  |
| 3+ yr | 378 (18%) | 18 (26%) | 61 (30%) | 106 (21%) | 98 (16%) | 76 (13%) | 19 (11%) |  |
| Cycling | 84 (3.9%) | 12 (17%) | 16 (8.0%) | 18 (3.6%) | 26 (4.2%) | 10 (1.7%) | 2 (1.2%) |  |
| **Daily dose (g/day, <=30g)** |  |  |  |  |  |  |  | **0.034** |
| Mean (SD) | 6.73 (3.21) | 5.75 (1.81) | 7.06 (3.48) | 6.79 (3.28) | 6.92 (3.24) | 6.59 (3.18) | 6.38 (2.96) |  |
| Median (Q1, Q3) | 5.00 (5.00, 10.00) | 5.00 (5.00, 5.00) | 5.00 (5.00, 10.00) | 5.00 (5.00, 10.00) | 5.00 (5.00, 10.00) | 5.00 (5.00, 8.00) | 5.00 (5.00, 8.00) |  |
| (Missing) | 7 | 0 | 0 | 3 | 1 | 2 | 1 |  |
| **Dose category** |  |  |  |  |  |  |  |  |
| Low (<3g) | 42 (2.0%) | 0 (0%) | 4 (2.0%) | 11 (2.2%) | 9 (1.5%) | 13 (2.2%) | 5 (2.9%) |  |
| Below common (3-4g) | 76 (3.5%) | 3 (4.3%) | 6 (3.0%) | 18 (3.6%) | 21 (3.4%) | 22 (3.8%) | 6 (3.5%) |  |
| Common (5g) | 1,281 (60%) | 49 (71%) | 115 (57%) | 296 (59%) | 354 (58%) | 357 (61%) | 110 (64%) |  |
| Elevated (6-10g) | 618 (29%) | 17 (25%) | 61 (30%) | 150 (30%) | 188 (31%) | 161 (28%) | 41 (24%) |  |
| High (11-30g) | 117 (5.5%) | 0 (0%) | 15 (7.5%) | 27 (5.3%) | 39 (6.4%) | 28 (4.8%) | 8 (4.7%) |  |
| Implausible (>30g) | 7 (0.3%) | 0 (0%) | 0 (0%) | 3 (0.6%) | 1 (0.2%) | 2 (0.3%) | 1 (0.6%) |  |
| **Creatine form (primary classification)** |  |  |  |  |  |  |  |  |
| Monohydrate only | 1,474 (69%) | 46 (67%) | 149 (74%) | 338 (67%) | 436 (71%) | 398 (68%) | 107 (63%) |  |
| Micronized only | 453 (21%) | 16 (23%) | 27 (13%) | 100 (20%) | 119 (19%) | 138 (24%) | 53 (31%) |  |
| Monohydrate + micronized | 116 (5.4%) | 5 (7.2%) | 16 (8.0%) | 38 (7.5%) | 31 (5.1%) | 22 (3.8%) | 4 (2.3%) |  |
| Non-monohydrate only (HCl / buffered / nitrate / blend / unsure) | 87 (4.1%) | 1 (1.4%) | 7 (3.5%) | 26 (5.1%) | 22 (3.6%) | 24 (4.1%) | 7 (4.1%) |  |
| Not reported | 11 (0.5%) | 1 (1.4%) | 2 (1.0%) | 3 (0.6%) | 4 (0.7%) | 1 (0.2%) | 0 (0%) |  |
| **Loading phase** |  |  |  |  |  |  |  | 0.2 |
| No | 1,587 (74%) | 50 (72%) | 151 (75%) | 391 (77%) | 450 (74%) | 428 (73%) | 117 (68%) |  |
| Not sure what a loading phase is | 208 (9.7%) | 7 (10%) | 13 (6.5%) | 39 (7.7%) | 60 (9.8%) | 63 (11%) | 26 (15%) |  |
| Yes | 346 (16%) | 12 (17%) | 37 (18%) | 75 (15%) | 102 (17%) | 92 (16%) | 28 (16%) |  |
| **Dosing frequency** |  |  |  |  |  |  |  |  |
| Only during training phases | 24 (1.1%) | 0 (0%) | 3 (1.5%) | 4 (0.8%) | 6 (1.0%) | 6 (1.0%) | 5 (2.9%) |  |
| Inconsistently | 29 (1.4%) | 2 (2.9%) | 3 (1.5%) | 6 (1.2%) | 5 (0.8%) | 10 (1.7%) | 3 (1.8%) |  |
| A few times per week | 119 (5.6%) | 3 (4.3%) | 17 (8.5%) | 36 (7.1%) | 24 (3.9%) | 31 (5.3%) | 8 (4.7%) |  |
| Once daily | 1,691 (79%) | 62 (90%) | 153 (76%) | 386 (76%) | 494 (81%) | 461 (79%) | 135 (79%) |  |
| Multiple times daily | 278 (13%) | 2 (2.9%) | 25 (12%) | 73 (14%) | 83 (14%) | 75 (13%) | 20 (12%) |  |

^1^ n (%); ^2^ NA; Kruskal-Wallis rank sum test; Pearson’s Chi-squared test

*Note.* Supplementation patterns stratified by age decade among the 2,178 men- and women-identified respondents. Cycling (non-ordinal duration) is shown as a separate level of Duration of use. Dose category labels follow Table 2 conventions (Low: <3 g/day; Below common: 3-4 g/day; Common: 5 g/day; Elevated: 6-10 g/day; High: 11-30 g/day; Implausible: >30 g/day excluded from regression models).

**Supplementary Table S2.** *IRT Item Parameters and Model Fit (2PL and GPCM)*

*Panel A.* 2PL binary discrimination (a) and difficulty (b) parameters.

| **Item** | **a (2PL, binary)** | **b (2PL, binary)** |
| --- | --- | --- |
| **Kidney damage** | 4.83 | -1.03 |
| **Hair loss** | 2.93 | -1.03 |
| **Steroid** | 1.77 | -2.41 |
| **Only lifters** | 1.17 | -3.39 |
| **Long-term unsafe** | 1.95 | -1.39 |

*Panel B.* GPCM polytomous item parameters (primary knowledge model).

| **Item** | **a (GPCM)** | **b1 (GPCM, incorrect to unsure)** | **b2 (GPCM, unsure to correct)** |
| --- | --- | --- | --- |
| **Kidney damage** | 3.40 | -2.22 | -1.08 |
| **Hair loss** | 2.73 | -2.78 | -1.08 |
| **Steroid** | 1.57 | -3.23 | -2.60 |
| **Only lifters** | 0.60 | -1.73 | -4.61 |
| **Long-term unsafe** | 1.80 | -3.29 | -1.47 |

*Panel C.* GPCM fit indices and marginal reliability.

| **Model** | **M2 (df)** | **p** | **RMSEA** | **SRMSR** | **TLI** | **CFI** | **Marginal reliability** |
| --- | --- | --- | --- | --- | --- | --- | --- |
| GPCM (5-item) | 6.58 (5) | 0.252 | 0.012 | 0.039 | 0.999 | 0.999 | 0.475 |

*Note.* Marginal reliability is modest (~0.48) by individual-difference standards; IRT-theta scores are best interpreted at the group level (e.g., as covariates in regression). Sum-score sensitivity analysis across primary outcomes is reported in Supplementary Table S6.

**Supplementary Table S3.** *Gender Differences in Creatine Knowledge Item Responses*

| **Statement** | **Men Correct** | **Men Unsure** | **Men Incorrect** | **Women Correct** | **Women Unsure** | **Women Incorrect** | **Fisher p** |
| --- | --- | --- | --- | --- | --- | --- | --- |
| Creatine causes kidney damage | 949 (86.2%) | 137 (12.4%) | 15 (1.4%) | 806 (74.8%) | 241 (22.4%) | 30 (2.8%) | <.001 |
| Creatine causes hair loss | 959 (87.1%) | 138 (12.5%) | 4 (0.4%) | 810 (75.2%) | 255 (23.7%) | 12 (1.1%) | <.001 |
| Creatine is a steroid | 1075 (97.6%) | 23 (2.1%) | 3 (0.3%) | 1004 (93.2%) | 69 (6.4%) | 4 (0.4%) | <.001 |
| Only beneficial for lifters | 1070 (97.2%) | 20 (1.8%) | 11 (1.0%) | 1035 (96.1%) | 27 (2.5%) | 15 (1.4%) | 0.383 |
| Long-term use is unsafe | 985 (89.5%) | 111 (10.1%) | 5 (0.5%) | 866 (80.4%) | 204 (18.9%) | 7 (0.6%) | <.001 |

*Note.* Distribution of response categories (Correct / Unsure / Incorrect) by gender identity for each of the five knowledge items in the primary battery. Fisher p computed by simulation (B = 10,000).

**Supplementary Table S4.** *Predictors of Above-Common Creatine Dosing (>5 g/day)*

| **Covariate** | **OR** | **95% CI** | **p-value** |
| --- | --- | --- | --- |
| **Age (years)** | 1.01 | 1.00, 1.02 | **0.021** |
| **Gender (women)** | 0.48 | 0.39, 0.59 | **<0.001** |
| **Duration (ordinal)** | 1.21 | 1.11, 1.32 | **<0.001** |
| **Activity (ordinal)** | 1.02 | 0.88, 1.17 | 0.8 |
| **Knowledge (IRT theta)** | 1.78 | 1.51, 2.10 | **<0.001** |
| **N goals endorsed** | 1.06 | 1.01, 1.11 | **0.020** |
| **Used loading phase** | 1.42 | 1.10, 1.84 | **0.008** |
| **N co-supplements** | 1.20 | 1.13, 1.27 | **<0.001** |

Abbreviations: CI = Confidence Interval, OR = Odds Ratio

*Note.* CI = confidence interval; OR = odds ratio. Daily dose excluded (tautological: outcome derived from dose). Loading phase reflects historical use of a loading protocol, not necessarily current loading behavior.

**Supplementary Table S5.** *Strongest Cross-Domain Network Edges*

| **Node 1** | **Node 2** | **Weight** |
| --- | --- | --- |
| Bloating [Side Effects] | Women [Demographics] | 0.273 |
| Cognition [Goals] | Cognitive [Free Text] | 0.253 |
| Long-term [Knowledge] | Continue [Outcomes] | 0.243 |
| Aging [Goals] | Age [Demographics] | 0.215 |
| Women [Demographics] | Duration [Supplementation] | 0.208 |
| Women [Demographics] | Dose [Supplementation] | 0.202 |
| Bloating [Side Effects] | Satisf. [Outcomes] | 0.194 |
| Performance [Goals] | Women [Demographics] | 0.186 |
| Only lifters [Knowledge] | Cognition [Goals] | 0.185 |
| Cognition [Goals] | Dose [Supplementation] | 0.182 |
| Dose [Supplementation] | Dosing [Free Text] | 0.173 |
| Long-term [Knowledge] | Satisf. [Outcomes] | 0.154 |
| Medical [Goals] | Dose [Supplementation] | 0.146 |
| Performance [Goals] | Benefit [Outcomes] | 0.144 |
| Performance [Goals] | Activity [Demographics] | 0.143 |

**Supplementary Table S6.** *Sensitivity Analyses: Knowledge Operationalization*

| **Predictor** | **IRT theta (primary)** | **Sum score** |
| --- | --- | --- |
| **Perceived benefit (logistic; OR)** |  |  |
| Gender (women) | 0.66 (0.55, 0.80)*** | 0.66 (0.55, 0.80)*** |
| Knowledge (per SD) | 1.30 (1.14, 1.48)*** | 1.29 (1.15, 1.44)*** |
| **Side effects (Poisson; PR)** |  |  |
| Gender (women) | 1.48 (1.15, 1.91)** | 1.49 (1.16, 1.92)** |
| Knowledge (per SD) | 1.01 (0.85, 1.19) | 1.04 (0.90, 1.19) |
| **Satisfaction (OLS; beta)** |  |  |
| Gender (women) | -0.03 (-0.18, 0.12) | -0.03 (-0.19, 0.12) |
| Knowledge (per SD) | 0.57 (0.46, 0.67)*** | 0.49 (0.40, 0.58)*** |
| **Positive experience composite (logistic; OR)** |  |  |
| Gender (women) | 0.68 (0.56, 0.82)*** | 0.68 (0.56, 0.82)*** |
| Knowledge (per SD) | 1.60 (1.40, 1.84)*** | 1.60 (1.41, 1.83)*** |

*Note.* Sensitivity analyses comparing the IRT theta primary specification against a standardized sum score (z-scored) across the four primary outcomes. ** indicates p < 0.05, *** indicates p < 0.001.

**Supplementary Table S7.** *Network Edge Stability Bootstrap*

| **Edge** | **Original weight** | **Boot mean** | **Boot 2.5%** | **Boot 97.5%** | **Inclusion %** | **Stability** |
| --- | --- | --- | --- | --- | --- | --- |
| Bloating–Women | 0.273 | 0.195 | 0.000 | 0.482 | 58.5 | Unstable |
| Cognition–Cognitive | 0.253 | 0.299 | 0.114 | 0.483 | 97.5 | Stable |
| Long-term–Continue | 0.243 | 0.279 | 0.000 | 0.616 | 76.0 | Moderate |
| Aging–Age | 0.215 | 0.219 | 0.139 | 0.299 | 100.0 | Stable |
| Women–Duration | 0.208 | 0.202 | 0.134 | 0.273 | 100.0 | Stable |
| Women–Dose | 0.202 | 0.194 | 0.126 | 0.261 | 100.0 | Stable |
| Bloating–Satisf. | 0.194 | 0.096 | 0.000 | 0.307 | 47.5 | Unstable |
| Performance–Women | 0.186 | 0.190 | 0.091 | 0.296 | 100.0 | Stable |
| Only lifters–Cognition | 0.185 | 0.163 | 0.000 | 0.437 | 63.5 | Unstable |
| Cognition–Dose | 0.182 | 0.192 | 0.123 | 0.255 | 100.0 | Stable |

*Note.* Nonparametric bootstrap (B = 200). Inclusion % = proportion of bootstrap samples where the edge weight was nonzero. Stability tier: Stable (Inclusion >= 80%), Moderate (65–79%), Unstable (<65%). CS-coefficient for strength centrality = 0.7 (>= 0.25 indicates interpretable centrality ordering; >= 0.50 indicates stable ordering).

**Supplementary Table S8.** *Model Specification Summary*

| **Model** | **Type** | **N** | **Outcome** | **Predictors** | **Correction** |
| --- | --- | --- | --- | --- | --- |
| **Primary multivariable models** |  |  |  |  |  |
| Perceived benefit (Table 3) | Logistic | 2,057 | Binary | Core | None |
| Side-effect prevalence (Table 5) | Mod. Poisson, HC0 robust SE | 2,057 | Binary | Core + loading phase | None |
| Satisfaction (Table 6) | OLS | 2,057 | 1-10 continuous | Core + side-effect history + n goals | None |
| Positive experience composite (Figure 6) | Logistic | 2,057 | Composite y/n | Core + n goals (sum-score sensitivity in Supp. Table S6) | None |
| **Pre-specified interaction** |  |  |  |  |  |
| Gender x dose interaction (extended model) | Logistic | 2,057 | Binary | Core + gender x dose; continuous predictors mean-centered | None |
| **Sub-models and exploratory** |  |  |  |  |  |
| Goal endorsement (Figure 3; 14 separate models) | Logistic | 2,178 | Binary (per goal) | Age (per yr) x gender | BH-FDR, 22 tests |
| Within-person goal-vs-benefit (Table 4) | McNemar exact-binomial | 805-1,476 | Paired y/n | Goal endorser x matched perceived-benefit (paired; no covariates) | None |
| 6 benefit-mode models (Figure S2; 6 separate models) | Logistic | 2,057 | Binary (per domain) | Core | BH-FDR, 42 tests |
| Above-common dosing (Supp. Table S4) | Logistic | 2,050 | >5 vs. <=5 g/day | Core (dose excluded; tautological) + loading + n goals | None |

*Note.* Core covariates = age (yr); gender (women/men); dose per 5 g; duration (ordinal); activity (ordinal); knowledge theta (IRT, SD units); n co-supplements. All binary outcomes combine ‘unsure’ with ‘no’. Knowledge theta has mean approximately 0 and SD approximately 1 in the regression sample. Analytic sample sizes are computed from the listwise-deletion complete-case sample, bounded by listwise deletion within each model. The within-person goal-vs-benefit McNemar tests are paired at the respondent level; per-domain N is the number of respondents endorsing each goal (range 805–1,476; see Table 4). The Above-common dosing model includes the loading-phase indicator as a predictor; N = 2,050 reflects listwise deletion of 7 respondents with missing loading-phase data relative to the N = 2,057 primary sample.
